# Dact2 Represses PITX2 Transcriptional Activation and Cell Proliferation through Wnt/beta-Catenin Signaling during Odontogenesis

**DOI:** 10.1371/journal.pone.0054868

**Published:** 2013-01-22

**Authors:** Xiao Li, Sergio Florez, Jianbo Wang, Huojun Cao, Brad A. Amendt

**Affiliations:** Department of Anatomy and Cell Biology, The University of Iowa, Iowa City, Iowa, United States of America; Institute of Neurology (Edinger-Institute), Germany

## Abstract

Dact proteins belong to the Dapper/Frodo protein family and function as cytoplasmic attenuators in Wnt and TGFβ signaling. Previous studies show that Dact1 is a potent Wnt signaling inhibitor by promoting degradation of β-catenin. We report a new mechanism for Dact2 function as an inhibitor of the canonical Wnt signaling pathway by interacting with PITX2. PITX2 is a downstream transcription factor in Wnt/β-catenin signaling, and PITX2 synergizes with Lef-1 to activate downstream genes. Immunohistochemistry verified the expression of *Dact2* in the tooth epithelium, which correlated with *Pitx2* epithelial expression. *Dact2* loss of function and *PITX2* gain of function studies reveal a feedback mechanism for controlling Dact2 expression. Pitx2 endogenously activates Dact2 expression and Dact2 feeds back to repress Pitx2 transcriptional activity. A Topflash reporter system was employed showing PITX2 activation of Wnt signaling, which is attenuated by Dact2. Transient transfections demonstrate the inhibitory effect of Dact2 on critical dental epithelial differentiation factors during tooth development. Dact2 significantly inhibits PITX2 activation of the Dlx2 and amelogenin promoters. Multiple lines of evidence conclude the inhibition is achieved by the physical interaction between Dact2 and Pitx2 proteins. The loss of function of Dact2 also reveals increased cell proliferation due to up-regulated Wnt downstream genes, cyclinD1 and cyclinD2. In summary, we have identified a novel role for Dact2 as an inhibitor of the canonical Wnt pathway in embryonic tooth development through its regulation of cell proliferation and differentiation.

## Introduction

The mouse tooth is an advantageous model to study organogenesis by analyzing molecular signaling networks that regulate cell differentiation and proliferation. The importance of signaling pathways including Wnts in the reciprocal interactions between oral epithelium and mesenchyme were proved in previous studies [1], [2]. The outer and inner dental epithelia are derived from oral epithelium, and are gradually differentiated into ameloblasts along the posterior-anterior axis. Several transcription factors including Pitx2, Dlx2, FoxJ1 and amelogenin (Amelx) have hierarchical expression during tooth development [3]. Together with the upstream signaling pathways, these mechanisms play critical roles in the dental crown and root formation [4]. As reported previously, Pitx2 is one of the earliest transcription markers observed during tooth development, and it is specifically restricted to the epithelium of the developing tooth. Pitx2 is regulated by the Wnt/β-catenin pathway and functions in the pathway by recruiting and independently interacting with Lef-1 and β-catenin to synergistically activate target genes, and many of these target genes are critical for tooth development [5], [6].

Dacts are intracellular proteins that can bind to many factors in both cytoplasmic and nuclear compartments. All members of the Dact family have N-terminal leucine zipper domains and C-terminal PDZ binding motifs [7], [8]. The orthologs of mouse Dact family members in xenopus, zebrafish and human are highly conserved in terms of gene structures. Studies have shown the conservation is also prominent at the functional level. In *Xenopus laevis*, xDact binds to Dvl through its C terminal PDZ binding motif [7], and targets β-catenin for destruction by the APC, Axin, and Gsk3α complex leading to down-regulation of β-catenin responsive genes. In zebrafish, Dact1 negatively regulates both canonical Wnt pathway and planar cell polarity (PCP) pathway (also known as non-canonical Wnt pathway) [9]. But Dact2 in zebrafish represses the PCP pathway [10] and TGFβ/Nodal pathway [11]. Mouse Dact1 degrades Dvl and antagonizes β-catenin dependent Wnt signaling in the same way as xDact in Xenopus [9]. Mouse Dact1 functions in the similar way as xDact by antagonizing the Wnt/β-catenin signaling, and intensively involved in the PCP pathway [12]. On the other hand, mouse Dact2 antagonizes TGFβ signaling [13], as well as Wnt/β-catenin signaling. However, mouse Dact2 inhibits Wnt/β-catenin signaling in a distinct manner from Dact1. Dact2 doesn't directly alter the level of β-catenin [14].

Wnt proteins belong to a family of ligands that are able to activate a receptor mediated signaling pathway [15], [16], [17]. Wnt signaling acting through the cytoplasmic scaffold protein dishevelled (Dvl) stabilizes β-catenin allowing it to enter the nucleus where it interacts with Pitx2 and Lef-1 to regulate gene expression [6], [18], [19]. Studies showed that Wnt/β-catenin signaling was highly conserved, and the precise control of Wnt signaling is required for normal tooth development. Inhibition of the Wnt/β-catenin pathway led to differentiation arrest of the dental epithelial cells and multiple other dental development defects [20], [21].

Studies on craniofacial and tooth development identified Dact2 as an important factor that is highly expressed in the dental epithelium and epithelial cells. Dact2 also has been shown to interact with various factors, including PKC, Dvl3, β-catenin and Lef-1, and Dact2 can form hetero/homo dimers with all three Dact family members. However, the molecular mechanisms of Dact2 function are still poorly understood. Dact2 is a substrate of kinases but whether the phosphorylation of Dact2 has any alteration of cellular function is unknown [22]. Dact2 mRNA localization has also been shown to be restricted in the dental epithelia including the cervical loops (stem cell niche) [23].

Because PITX2 interacts with β-catenin and Lef-1 to regulate gene expression we asked if Dact2 interacted with PITX2 to modulate PITX2 transcriptional activity. Furthermore, we hypothesized that Dact2 regulated β-catenin activity in the dental epithelium and subsequently tooth development. The *Dact2* null mice were analyzed for tooth developmental, cell proliferation and/or differentiation defects. These studies reveal a role for Dact2 in modulating Wnt/β-catenin signaling activity through PITX2.

## Materials and Methods

### Histology and fluorescent immunohistochemistry

All animals were housed in the Program of Animal Resources of the Institute of Biosciences and Technology, and were handled in accordance with the principles and procedure of the Guide for the Care and Use of Laboratory Animals. The Texas A&M Health Science Center, Institutional Animal Care and Use Committee approved all experimental procedures. The *Dact2* null mice (NM_172826) were obtained from the Texas Institute for Genomic Medicine and the *Dact2* gene was inactivated using the gene trap insertion method. The insertion completely inactivated the *Dact2* gene and no Dact2 protein was produced in the mutant mice. Murine embryos were used for histology and fluorescent immunohistochemistry (FIHC). Samples were fixed in 4% paraformaldehyde, dehydrated and embedded in paraffin wax. Sections were cut (7 μm) and stained with Hematoxylin and Eosin. Sections for immunohistochemistry were rehydrated and treated with 10 mM Sodium Citrate solution for 15 min at a slow boiling state for antigen retrieval. Subsequently sections were incubated with 10% goat serum-PBST for 30 min at the room temperature, followed by overnight incubation with specific primary antibody at dilution of 1∶500 at 4°C. After the incubation the slides were treated with FITC labeled secondary antibody (Invitrogen) at a concentration of 1∶300 for 30 min. Each antibody incubation was followed by 3–6 PBST (phosphate-buffered saline with tween) washes. β-catenin antibody was purchased from Santa Cruz Biotechnology. Nuclear counter staining was performed using a DAPI containing mounting solution after the final wash (Vector Laboratories).

### Fluorescent immunocytochemistry

Cells were seeded on microscope glass cover slips in 60 mm dishes 24 h prior to fixation. Fixation was done by incubating the cover slips in ice-cold acetone for 5 min at 4°C. Fixed cells were washed with PBST for 5 min twice. Subsequently the cover slips were incubated in 10% normal goat serum-PBST 30 min at room temperature, and then in specific primary antibodies at 4°C. After overnight incubation, cells were rinsed by PBST for 3 times, 5 min each. Then the cells were incubated with FITC labeled secondary antibody for 30 min at 37°C. Finally the cells were washed with PBST for 3 times, 5 min each, and counter stained by DAPI containing mounting solution.

### Expression and reporter constructs

Expression plasmids containing the cytomegalovirus (CMV) promoter linked to the PITX2A cDNA were constructed in pcDNA-3.1-MycHisC (Invitrogen) [18], [24], [25]. β-catenin S37A expression plasmid has been previously described [6]. Mouse *Dact2* cDNA (NM_172826) was cloned and constructed into pcDNA-3.1-MycHisC (Invitrogen) backbone, under control of the CMV promoter. Dlx2 and amelogenin promoters in pTK-Luc have been previously described [3], [4]. 10 kb Dact2 promoter was cloned in pTK-Luc plasmid. The 66 bp Dact2 enhancer reporter was constructed by cloning a 66 bp DNA segment of Dact2 promoter containing the Pitx2 binding site at (−6172 to −6106 bp) into minimal TK-Luc reporter in tandem. Mutant reporter was constructed with the same tandem flanking promoter sequence, except the Pitx2 binding motif GGATTA (−6142 to −6136 bp) was mutated into scrambled motif AGTTCG. All constructs were confirmed by DNA sequencing. All plasmids were double-banded CsCI purified.

### Cell culture, transient transfections, luciferase and β-galactosidase assays

MDPC, LS-8 cells [26], CHO cells and HEK293FT cells were cultured in DMEM supplemented with 5% FBS, 5% BGS and penicillin/streptomycin and transfected by electroporation. Cells were fed and seeded in 60 mm dishes 24 hours prior of transfection. Cells were resuspended in PBS and mixed with 2.5 μg of expression plasmids, 5 μg of reporter plasmid and 0.2 μg of SV-40 β-galactosidase plasmid. Electroporation was performed at 380 v and 950 μF (Gene Pulser XL, Bio-Rad). Transfected cells were incubated for 24 h (unless otherwise indicated) in 60 mm culture dishes and fed with 10% FBS and DMEM and then lysed and assayed for reporter activities as well as protein content by Bradford assay (Bio-Rad). Luciferase level was measured using the luciferase assay kit (Promega). β-galactosidase was measured using the Galacto-Light Plus reagents (Tropix Inc.). All luciferase activities were normalized to β-galactosidase activity.

### Western blot assays

Approximately 15 μg of transfected cell lysates were analyzed in Western blots. Following SDS gel electrophoresis, the proteins were transferred to PVDF filters (Millipore), immunoblotted and detected using specific antibodies and ECL reagents (GE HealthCare). Endogenous Dact2 expression was detected with polyclonal rabbit antibody (ProSci, Inc.) GAPDH was detected with polyclonal mouse antibody (Millipore). β-tubulin was detected by polyclonal rabbit antibody (Santa Cruz Biotechnology). Myc tag was detected by polyclonal mouse antibody (Invitrogen). Quantification of band intensity was performed by ImageMeter software (Flashscript.biz). Intensity of each band was normalized with corresponding loading control (i.e. GAPDH), and then converted as relative fold value of the intensity of protein of interest in the first lane of the blot. ±SEM were calculated based on at least 3 different blots.

### Chromatin Immunoprecipitation (ChIP) assay

The ChIP assays were performed as previously described using the ChIP Assay Kit (Upstate) with the following modifications [6], [27]. LS-8 cells were plated in 60 mm dishes and fed 24 h prior to the experiment, harvested and plated in 60 mm dishes. Cells were cross-linked with 1% formaldehyde for 10 min at 37°C. All PCR reactions were done with an annealing temperature of 58°C. Specific primers for amplifying the Pitx2 binding site in the *Dact2* promoter were as follow: sense: 5′- ACTAACGGGAGCCCTGACAT -3′ and antisense: 5′ -GGAGGCATTTTTCTCAATGG-3′. All the PCR products were evaluated on a 2% agarose gel in TBE for expected size (292 bp) and confirmed by sequencing. As controls the primers were used without chromatin, normal rabbit IgG was used replacing the specific primary antibody to reveal non-specific immunoprecipitation of the chromatin. The primers for amplifying the *Msx2* promoter were as follow: sense: 5′ -AAGGGAGAAAGGGTAGAG- 3′ and antisense: 5′ -CCCGCCTGAGAATGTTGG-3′. The expected product size was 273 bp. The primers for amplifying the non-conserved binding motif at -3719 bp on *Dact2* promoter were as follow: 5′ -CCTCTGGAAGCAGGAGAGTG- 3′ and antisense: 5′ -CACTCTCCTGCTTCCAGAGG-3′. The expected product size was 236 bp. The primary antibody used in this assay was polyclonal rabbit Pitx2 antibody (Capra Science). Evolutional conservation analysis was performed using online tool (http://ecrbrowser.dcode.org/), [28].

### Immunoprecipitation Assay

LS-8 oral epithelial cells were used to demonstrate endogenous Dact2 and Pitx2 interaction. Cells were harvested and disrupted by repeated aspiration through a 25-gauge needle. Cellular debris was pelleted at 4°C. An aliquot of lysate was saved for analysis as input control. The supernatant was transferred to a fresh 1.5-ml microcentrifuge tube on ice and precleared using the mouse IgG. Precleared lysate was incubated with protein A/G-agarose beads for 1–2 h at 4°C. After a brief centrifugation, supernatant was transferred to a new tube, and immunoprecipitation was performed with rabbit Pitx2 antibody (Capra Science). The supernatant was incubated with protein A/G-agarose beads at 4°C for overnight. Immunoprecipitates were collected by brief centrifugation and washed 3 times with PBS and resuspended in 15 μl of H_2_O and 3 μl of 6X SDS loading dye. Samples were boiled for 5 min and resolved on a 12% polyacrylamide gel. Western blotting was performed with anti-Dact2 antibody and HRP-conjugated antibody to detect immunoprecipitated proteins.

### GST Pulldown Assays

GST-PITX2A-FL (full length), GST-PITX2A-HD (homeodomain), GST-PITX2A-NΔ38 and GST-PITX2A-C173 fusion proteins were expressed in bacteria, purified and immobilized on Glutathione-Sepharose beads. Protein binding beads were suspended in binding buffer (20 mM HEPES, pH 7.5, 5% glycerol, 50 mM NaCl, 1 mM EDTA, 1 mM DTT with 1% milk and 400 μg/ml ethidium bromide). Purified bacterial expressed Dact2 proteins were added to 10–30 μg of immobilized GST fusion proteins in a total volume of 100 μl and incubated for 30 min at 4°C. The beads were pelleted and washed 5 times with 200 μl of binding buffer. The bound proteins were eluted by boiling in SDS sample buffer and separated on a 12% SDS-polyacrylamide gel. Immunoblotting detected Dact2 protein using Dact2 antibody (ProSci) and ECL reagents from GE Healthcare.

### Cell proliferation assays

MEF cells were harvested from E13.5 littermates and subjected to cell proliferation assay before passage 3. 1.5×10^5^ cells of each line were seeded in 60 mm plates on day 0. Cells were then trypsinized and counted after 24, 48, 72 and 96 hours by a Coulter Z1 cell counter (Beckman Coulter, Inc). Experiments were run in 4 replicates.

### Real-time PCR assays

RNA extraction was performed using RNeasy Mini kit from Qiagen. RT-PCRs were performed using iScript Select cDNA synthesis kit from BioRad. Real-time PCRs were performed using iQ SYBR Green Supermix kit, and all Ct values were normalized by β-actin level. Both isoforms of endogenous Dact1 were measured using forward (5′- AGCCTCGTGCAGAAGAAAAC -3′) and reverse (5′- CAGAGCCAACCTCTTGCTTT -3′) primers. Dact2 was measured using forward (5′- GGCTGACGGGCATGTTC -3′) and reverse (5′- CCCCACGTCAGCTGGAA -3′) primers. Dact3 was measured using forward (5′- GAGCTGAGACCTGCTCATCC -3′) and reverse (5′- GAGCTGAGACCTGCTCATCC -3′) primers. Primers to measure Ccnd2 were forward (5′- GTTCTGCAGAACCTGTTGAC -3′) and reverse (5′- ACAGCTTCTCCTTTTGCTGG -3′). Primers for Ccnd1 and c-Myc were previously described [29]. All PCR products were examined by melt curves and sequenced.

### RNA interference

Short-hairpin (sh) RNA plasmids carrying sequence targets on the *Dact2* mRNA, 5′-TGGATGTGAGCAGGTCTTCTT-3′ was used to transfect LS-8 cells to specifically knock down *Dact2* mRNA. This shRNA sequence was previously described and proven effective [14]. Control shRNA was targeting firefly luciferase and did not match any mouse cDNA (by a BLAST search). Transfection of cells was performed using 5 μg plasmid per 1×10^6^ cells, and cultured for 48 hours before harvesting.

### Statistical Analysis

Two-tailed unpaired Student's t test was used to determine the difference between two sets of values. Error bars were expressed as mean ± SE. All experiments were repeated at least thrice.

### Ethics Statement

All animals were housed at the Institute of Biosciences and Technology under the care of the Program of Animal Resources, and were handled in accordance with the principles and procedure of the Guide for the Care and Use of Laboratory Animals. All experimental procedures were approved by the Texas A&M Health Science Center, Institutional Animal Care and Use Committee. Protocol number 09001, mouse models for tooth development.

## Results

### Dact2 is expressed in the dental epithelium

To characterize factors in dental development, we extracted tooth germ total RNA from epithelium and mesenchyme of P0 wild type mice and genomic microarrays were employed to distinguish epithelial from mesenchyme specific factors. Several epithelial markers such as Pitx2 and Enamlin (Enam) were highly expressed in dental epithelium, and mesenchyme markers such as Bmp2 and Pax9 were highly expressed in dental mesenchyme. Interestingly, the expression of Dact2 was significantly higher (4.45 fold) in dental epithelium compared to mesenchyme (Fig. S1A). Western blots confirmed endogenous Dact2 expression in the dental epithelial-like LS-8 cell line, in contrast with the mesenchyme odontoblast-like MDPC cells (Fig. S1B). A previous report revealed 3 members of the *Dact* gene family have correlated expression pattern in various tissues, and they form homo/hetero dimers among each other [22]. To determine whether *Dact1* and *Dact3* are co-expressed in the dental epithelium, we extracted RNA from LS-8 cells and found *Dact2* and *Dact1* have high expression levels, while *Dact3* transcripts are barely detectable (Fig. S1C). In-situ studies have shown abundant *Dact2* transcripts restricted in both incisor and molar epithelia, but *Dact1* and *Dact3* transcripts are present in the mesenchyme [23]. *Dact1* and *Dact3* are highly expressed in the dental mesenchyme MDPC cells (Fig. S1C).

**Figure 1:**
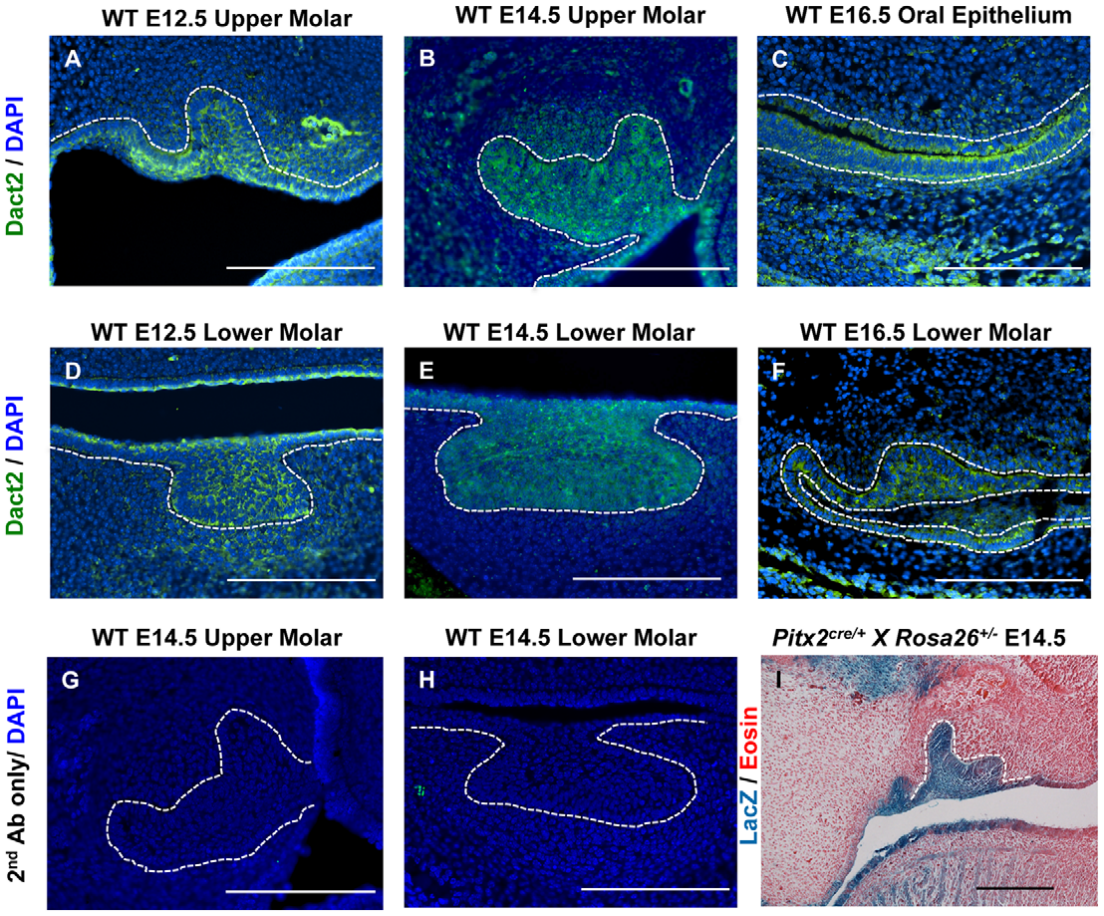
*Dact2* expression in dental and oral epithelia. (**A–F**) Endogenous Dact2 protein was stained using a Dact2 antibody and secondary FITC conjugated antibody and nuclei were stained by DAPI on wild type mouse embryos. (**A**) E12.5 upper molar tooth germ, (**B**) E14.5 upper molar tooth germ, (**C**) E16.5 oral epithelium, (**D**) E12.5 lower molar tooth germ, (**E**) E14.5 lower molar tooth germ, and (**F**) E16.5 first lower molar all shows epithelial expression of Dact2. (**G** and **H**) molar germs at E14.5 stained without Dact2 primary antibody as negative controls. (**I**) LacZ staining with eosin counter staining on E14.5 *Pitx2^cre/+^X Rosa26^+/−^* mice showed Pitx2 highly expressed cell linages in the upper molar bud, indicating overlapping expression of Dact2 with Pitx2 at the same developmental stages. White dotted lines indicate the mesenchyme-epithelium boundaries. Scale bar represents 100 μm.

To demonstrate Dact2 expression during odontogenesis, we performed immunohistochemistry using sagittal sections of El2.5, E14.5 and E16.5 upper and lower molars and oral epithelium. Fluorescent immunochemical staining was performed on a series of sections across the whole tooth germ to avoid positional bias and secondary antibody only staining was performed in parallel to ensure antibody specificity. Dact2 protein was specifically expressed in the dental epithelium and adjacent oral epithelium during incisor and molar development, consistent with the in situ hybridization results in previous reports (Fig. 1A–H) [8], [23].

### Dact2 expression correlates with Pitx2

Since Dact2 expression was observed in the dental epithelium, we asked whether it correlates with the expression of Pitx2. Pitx2 is one of the earliest transcription factors to initiate tooth formation, and strongly interacts with β-catenin to synergistically activate Wnt downstream genes [6]. Epithelial specific Pitx2 expression was observed at E14.5 using the *Pitx2^cre/+^Rosa26^+/−^*mouse (Fig. 1I and Fig. S2), and was previously shown by IHC [30]. These data demonstrate that Pitx2 and Dact2 are co-expressed in the dental epithelium during development. We next asked if Dact2 was part of the Wnt/Pitx2 transcriptional activator mechanism.

### PITX2 activates Dact2 expression

**Figure 2:**
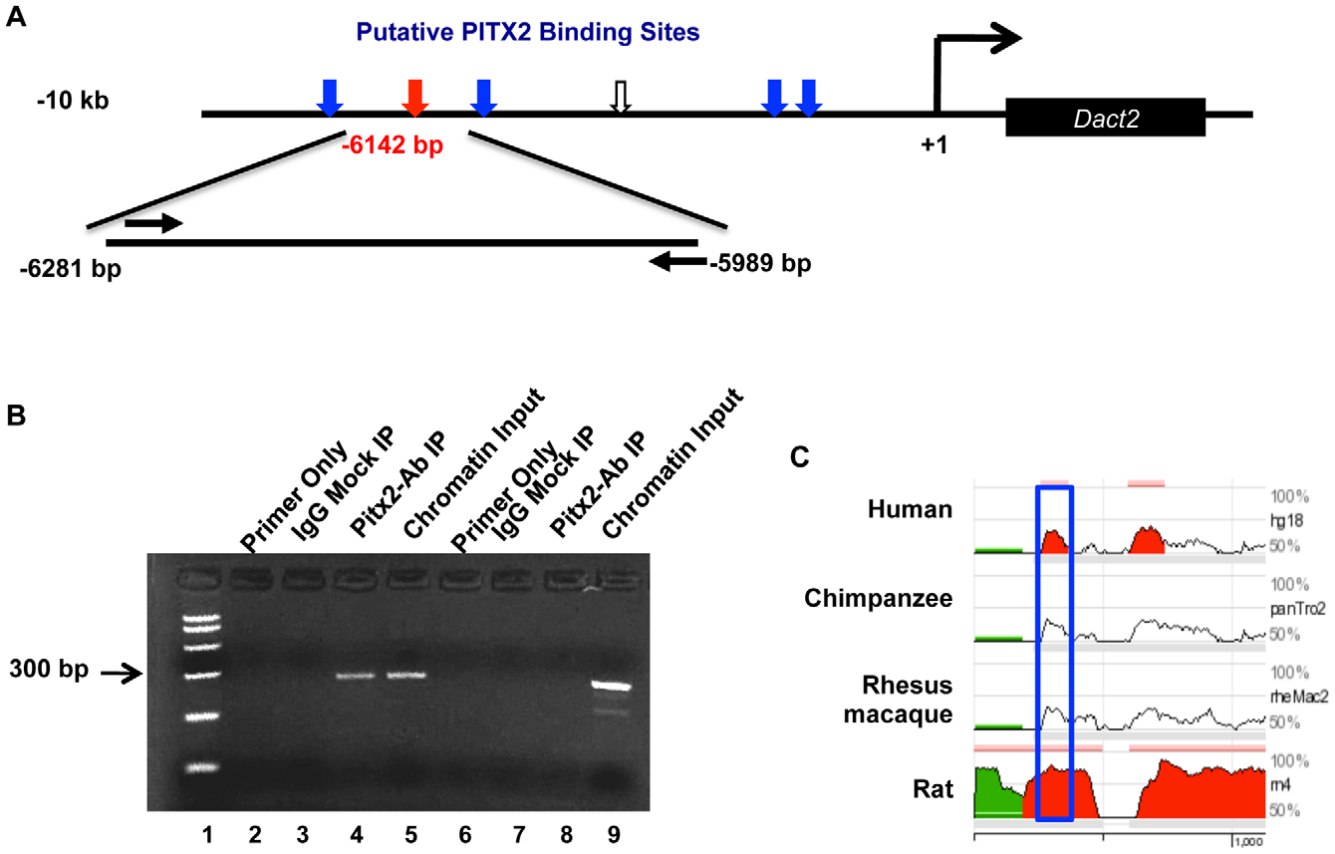
Endogenous Pitx2 binds to a conservative region on the *Dact2* promoter. (**A**) Schematic of *Dact2* 10 kb promoter with six PITX2 binding motifs (TAATCC) indicated by arrowheads. The red arrowhead indicates the site verified by ChIP assay. The location of the sense primer and the antisense primer are shown for amplification of the immunoprecipitated chromatin. Blue arrowheads are putative binding sites with less conservation. The white arrowhead indicate a non-conserved Pitx2 binding motif that we tested in ChIP experiment as negative control shown in Figure S3. (**B**) Endogenous ChIP assay was performed in LS-8 cells. Lane 1 contains the PCR marker. Lane 2 shows the *Dact2* primers-only control. Lane 3 is the immunoprecipitation using normal rabbit IgG and *Dact2* primers. Lane 4 is the Pitx2 immunoprecipitated chromatin amplified using the specific *Dact2* promoter primers. Lane 5 is the chromatin input amplified using the Dact2 primers. Lane 6 shows the *Msx2* promoter primers-only control. Lane 7 is the immunoprecipitation using normal rabbit IgG and *Msx2* primers. Lane 8 is the Pitx2 immunoprecipitated chromatin amplified using the specific *Msx2* promoter primers. Lane 9 is the chromatin input amplified using the *Msx2* primers. The amplified region of *Msx2* promoter is −632 to −359 bp relative to transcription start site. All PCR products were sequenced to confirm their identity. (**C**) The PITX2 binding element on mouse *Dact2* promoter verified by ChIP was mapped to a highly conserved (>70%) region among Mouse, Human, Chimpanzee, Rhesus macaque and Rat. The blue box indicates the PCR amplified region on Dact2 promoter in (**B**).

We analyzed the sequence of the 5′ flanking region of the *Dact2* gene and found several potential Pitx2 binding sites. After an evolutional conservation screening a putative binding site at -6142 bp was found with a high degree of conservation among mouse, rat, human and chimp (Fig. 2A, C). Chromatin immunoprecipitation (ChIP) assays performed in LS-8 cells demonstrate endogenous Pitx2 binding to this site on the *Dact2* promoter (Fig. 2B). A set of primers flanking this Pitx2 binding site (GGATAA) were able to amplify the *Dact2* promoter from chromatin input (Fig. 2B, lane 5), as well as from the Pitx2 immunoprecipitated chromatin (lane 4), demonstrating Pitx2 specifically binds to the element in the *Dact2* promoter. A PCR with DNA pulled down by normal IgG was examined as a negative control (Fig. 2B, lane 3). Since Pitx2 does not regulate Msx2, a negative control experiment was done in parallel using the same ChIP DNA pulled down by Pitx2 antibody and control IgG to amplify *Msx2* promoter region with specific *Msx2* primers. (Fig. 2B, lane 8). In addition, we performed another control experiment by testing a *Dact2* promoter fragment containing a putative binding site (−3753 to −3517 bp) with no significant conservation (Fig. S3A). The result shows no enrichment in Pitx2 antibody pull down DNA (Fig. S3B).

**Figure 3:**
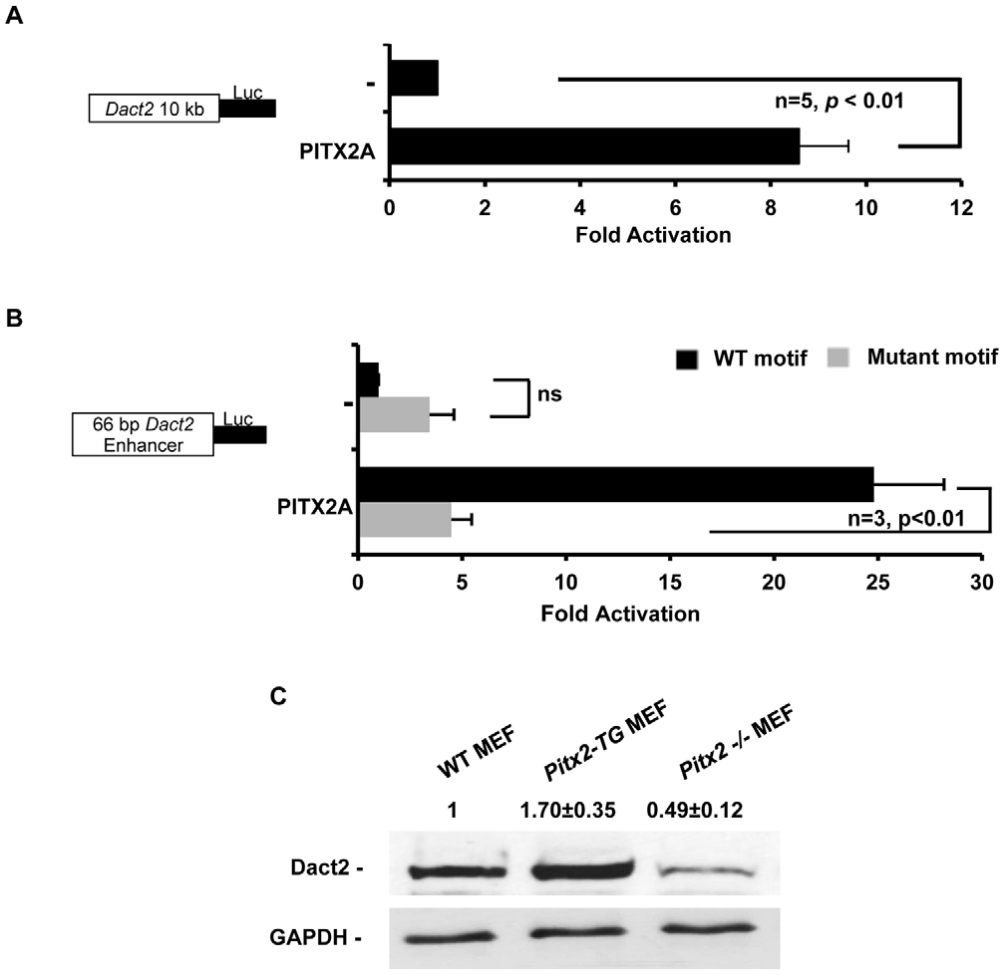
PITX2 activates Dact2 expression. (**A**) CHO cells were co-transfected with *CMV-PITX2A* expression plasmids and luciferase reporter driven by *Dact2* 10 kb promoter. Empty *CMV-PITX2A* expression plasmids were transfected in parallel as a negative control. All transfections included the SV-40-β-galactosidase reporter to control the transfection efficiency. Cells were incubated for 24 hrs and then assayed for luciferase and β-galactosidase activities. (**B**) Luciferase reporters driven by a duplicated 66 bp DNA segment of *Dact2* promoter flanking the Pitx2 binding site in Fig. 2A at (−6172 to −6106) was co-transfected with or without *CMV-PITX2A* overexpression plasmid in CHO cells. A similar reporter with the mutated Pitx2 binding motif was transfected in parallel as control. All luciferase activities are shown as mean-fold activation compared with the *Dact2* promoter plasmid co-transfected with empty CMV expression plasmid (±SEM from five independent experiments). (**C**) E14.5 Embryos from *Pitx2^−/−^, Pitx2* transgenic and wild type mice were harvested to generate MEF cells. These MEFs were lysed and analyzed by Western blots to show endogenous Dact2 expression levels. GAPDH expression was probed as loading controls. Protein band intensities were quantified and shown as relative value ±SEM.

To verify whether this binding is functional, we performed transient co-transfection in cells with PITX2 expression and a luciferase expression plasmid driven by the 10 kb *Dact2* promoter. Cells transfected with PITX2 activated the *Dact2* promoter about 8-fold (Fig. 3A). A 66 bp DNA segment of *Dact2* promoter containing the PITX2 binding site in Fig. 2A at (−6172 to −6106 bp) was cloned into TK-Luc reporter in tandem. A similar reporter was constructed with the same tandem flanking promoter sequence, except the binding motif GGATTA (−6142 to −6136) in this reporter was mutated into scrambled motif AGTTCG. Luciferase assays in Fig. 3B conducted on CHO cells transfected with PITX2A and each of these two reporters showed a loss of PITX2 activation when the binding motif was mutated (Fig. 3B).

Furthermore, endogenous Dact2 expression was increased in *PITX2*C transgenic mouse embryonic fibroblast (MEF) cells compared to wild type MEF cells (Fig. 3C). Furthermore, Dact2 expression was very low in *Pitx2* knockout MEF cells. These data strongly suggest that endogenous Pitx2 activates Dact2 expression.

### Dact2 represses PITX2 transcriptional activity

**Figure 4:**
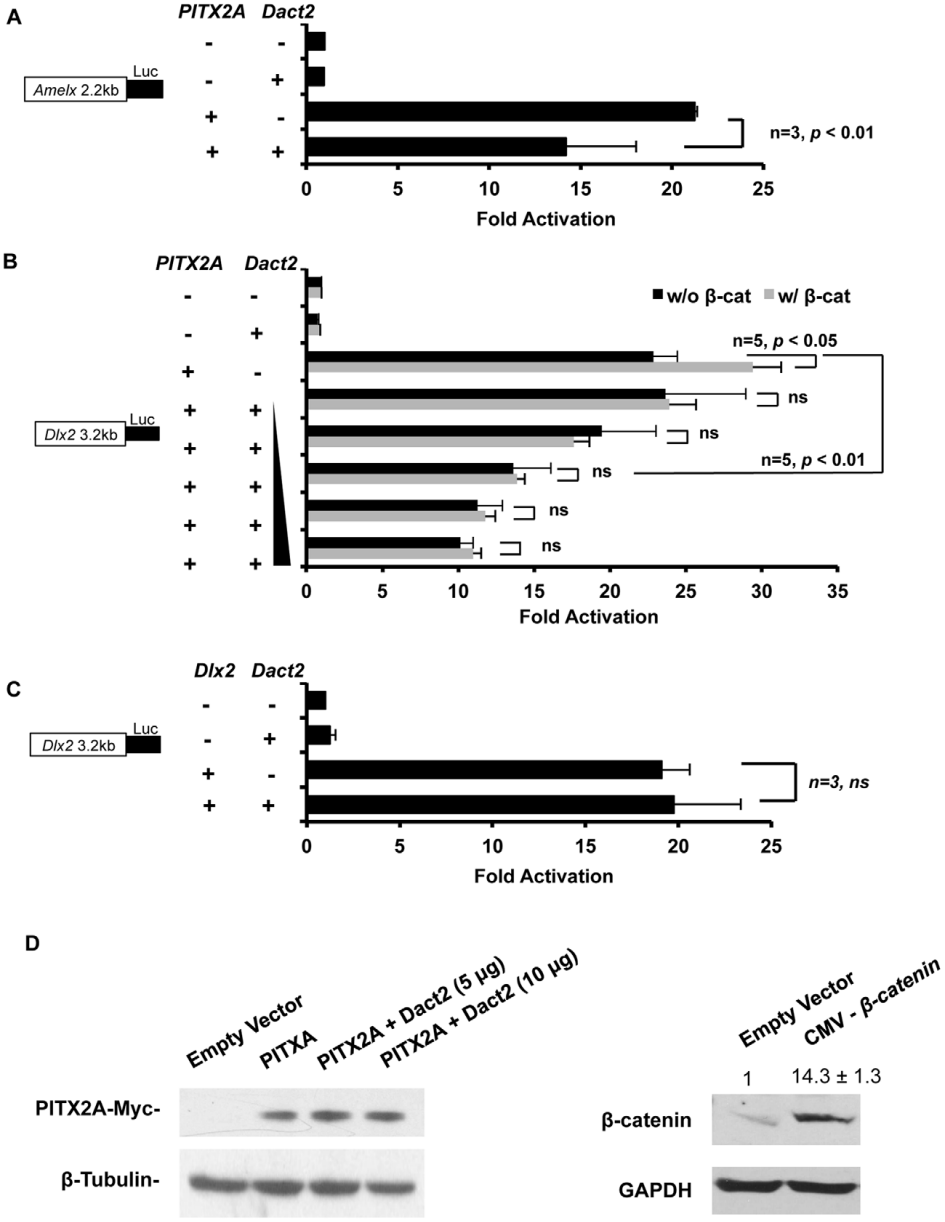
Dact2 attenuates PITX2 transcription activity. (**A**) CHO cells were transfected with *CMV-PITX2A, CMV-Dact2* and luciferase reporter driven by *Amelx* 2.2 kb promoter. Empty CMV expression plasmids were transfected in parallel as a negative control. (**B**) CHO cells were transfected with combinations of *CMV-PITX2A, CMV-β-catenin, CMV-Dact2* and luciferase reporter driven by *Dlx2* 3.2 kb promoter. Empty CMV expression plasmids were transfected in parallel as a negative control. The titration gradient of transfected *Dact2* expression plasmids are from 0.5 μg to 8 μg in 2-fold increment. The luciferase activities were normalized by co-transfected β-galactosidase and ±SEM was from five independent experiments. (**C**) Dlx2 was transfected instead of PITX2A to show Dact2 attenuation is specific to PITX2 transcription activity. All luciferase activities were normalized by co-transfected β-galactosidase and ±SEM were from at least three independent experiments. (**D**) Dact2and PITX2A transfected CHO cells were lysed and analyzed by Western blot, probing for transfected PITX2A. β-catenin expression is shown by Western blot in transfected cells. β-tublin was probed as loading control.

To understand the function of Dact2, cell transfections were performed with Dact2 and PITX2 over expression plasmids and luciferase reporter constructs under the control of Dlx2 and amelogenin promoters. Dlx2 is an ameloblast differentiation marker under Pitx2 regulation in the transcriptional hierarchy of tooth development [3], [31]. Amelogenin (Amelx) is a structural protein that contributes to the enamel formation during the late stage of tooth development, which is also under transcriptional control of Pitx2. The luciferase reporters driven by Amelx and Dlx2 promoters were analyzed for Dact2 function. PITX2A activates the Amelx promoter at ∼22-fold and Dact2 alone does not activate the Amelx promoter in transfected cells (Fig. 4A). However, Dact2 represses PITX2 activation of the Amelx promoter, from 22-fold to 14-fold activation (Fig. 4A).

Because Dact2 is involved in Wnt/β-catenin signaling we asked if Dact2 repression of PITX2 transcriptional activity was modulated by β-catenin. PITX2 and β-catenin interact to increase the activity of PITX2 and co-expression of both activate the Dlx2 promoter at 30-fold whereas co-expression of Dact2 and β-catenin did not activate the Dlx2 promoter in LS-8 cells (Fig. 4B). The Dact2 expression plasmid was titrated against constant amounts of PITX2 and β-catenin plasmids (2.5 μg) and the Dlx2 reporter (5 μg). As the Dact2 plasmid concentration was increased from 0.5 μg, 1 μg, 2 μg, 4 μg and 8 μg, this correlated with increased repression of PITX2 transcriptional activity in the presence of β-catenin (Fig. 4B). Dose dependent inhibitory effect of Dact2 also exists when β-catenin is not ectopically expressed. Thus, Dact2 inhibition of PITX2 is not β-catenin dependent and is not affected by the interaction between β-catenin and PITX2. Dact2 does not repress Dlx2 activation of the Dlx2 promoter indicating that the repression by Dact2 is specific to PITX2 (Fig. 4C).

To rule out the possibility that PITX2 was degraded after over expression of Dact2, Western blots were performed using PITX2A and Dact2 co-transfected LS-8 cells. Transfected PITX2A levels remained constant with different amounts of transfected Dact2 (transfected PITX2 was observed using the Myc antibody, top blot, Fig. 4D). β-catenin over expression is shown by Western blot (Fig. 4D).

### Dact2 is a potent modulator of Wnt/β-catenin signaling

**Figure 5:**
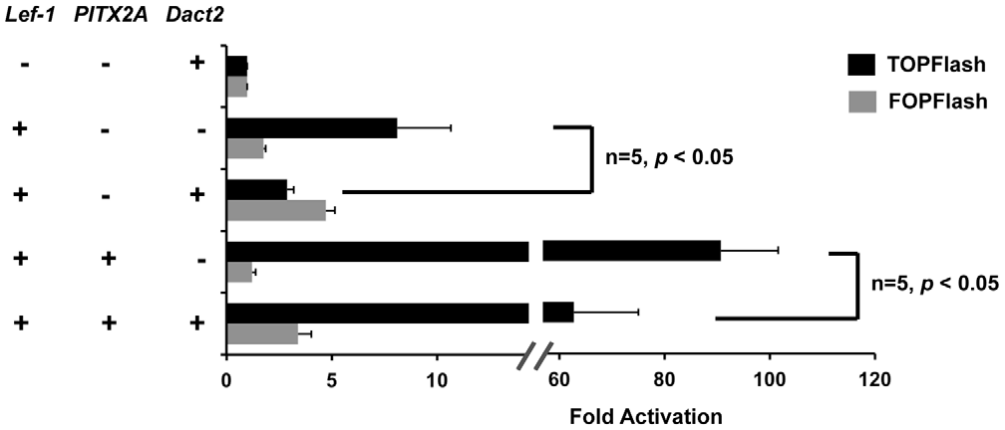
Dact2 is a potent inhibitor of Wnt/β-catenin signaling. 293FT cells were transfected with *CMV-Lef-1, CMV-PITX2A, CMV-Dact2* overexpression plasmids and TOPFlash reporters. In parallel experiments FOPFlash reporter were transfected as negative control. All luciferase activities were normalized by co-transfected β-galactosidase and ±SEM were from five independent experiments.

Pitx2 interacts with β-catenin and Lef-l independently and synergistically activates the target genes of Wnt/β-catenin signaling by recruiting β-catenin and Lef-l to the same transcriptional activation complex [5], [6]. We employed the Topflash reporter plasmid, which is a widely used indicator of Wnt/β-catenin signal transduction to determine the role of Dact2 in regulating Wnt/β-catenin activation of transcription. The Topflash reporter contains the luciferase gene driven by multiple TCF/LEF binding sites and the Fopflash plasmid contains point mutations in the TCF/LEF binding sites, which abolish binding of Lef-1 and was used as a negative control. Transfected Lef-1 (2.5 μg) activated the Topflash reporter (5 μg) by about 8-fold, and transfected Dact2 (2.5 μg) strongly repressed this activation (Fig. 5). Co-transfection of PITX2A and Lef-1 activated Topflash at 90-fold and addition of Dact2 repressed this activation to ∼60-fold (Fig. 5). Dact2 expression repressed the PITX2 and Lef-1 synergistic activation of Topflash by 30%. These results indicate that Dact2 represses the activation of tooth development factors and the Wnt/β-catenin pathway in general by interfering with PITX2 and Lef-1 transcriptional activity.

This repression by Dact2 can be rescued in a dose dependent manner by introducing Dact2 shRNA (Fig. S4A). This result verifies that repression of Topflash is specificity due to Dact2 function. We noticed the mild activation on Fopflash by overexpressing Dact2. But this activation cannot be reversed by introducing Dact2 shRNA (Fig. S4B).

### Dact2 is both cytoplasmic and nuclear localized and co-localizes with Pitx2 in the nucleus

**Figure 6:**
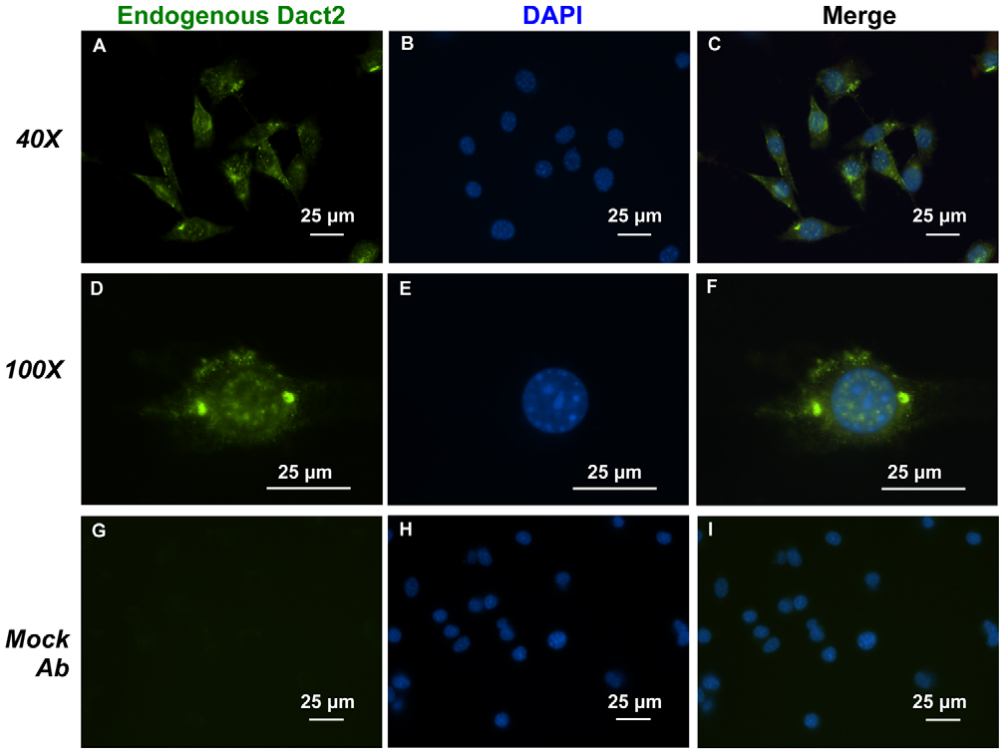
Dact2 protein localizes to the cytoplasm and nuclear compartments in LS-8 cells. Endogenous immunofluorescence staining on LS-8 cells was performed. (**A**) Dact2 protein was probed by Dact2 primary antibody and labeled with FITC. (**B**) Nuclei were stained by DAPI. (**C**) Merged signals of FITC and DAPI. (**D, E** and **F**) Single cell was viewed under 100X objective. (**G, H** and **I**) Parallel experiments using normal rabbit IgG as mock primary antibody to show secondary antibody specificity. All scale bars represent 25 μm.

The Dact1 protein shuttles between the nucleus and cytoplasm [32]. Because Dact2 and Dact1 proteins have high homology, Dact2 may also have this property. We performed endogenous immunofluorescence staining on LS-8 cells. Dact2 labeled with FITC appears in both nuclear and cytoplasm compartments in discrete loci (Fig. 6A–F). Evidence from Xenopus showed xDact proteins are present in both cytoplasm and nuclear compartments [7]. An IgG mock control staining was used in parallel to demonstrate the specificity of Dact2 staining (Fig. 6G–I). LS-8 cells transfected with Dact2 shRNA were also stained for Dact2 protein. Compared to non-transfected LS-8 cells (Fig. S5A) and LS-8 cells transfected with scrambled shRNA (NC shRNA) (Fig. S5B), the overall level of Dact2 expression was significantly reduced by Dact2 shRNA (Fig. S4B), demonstrating the specificity of Dact2 staining.

**Figure 7:**
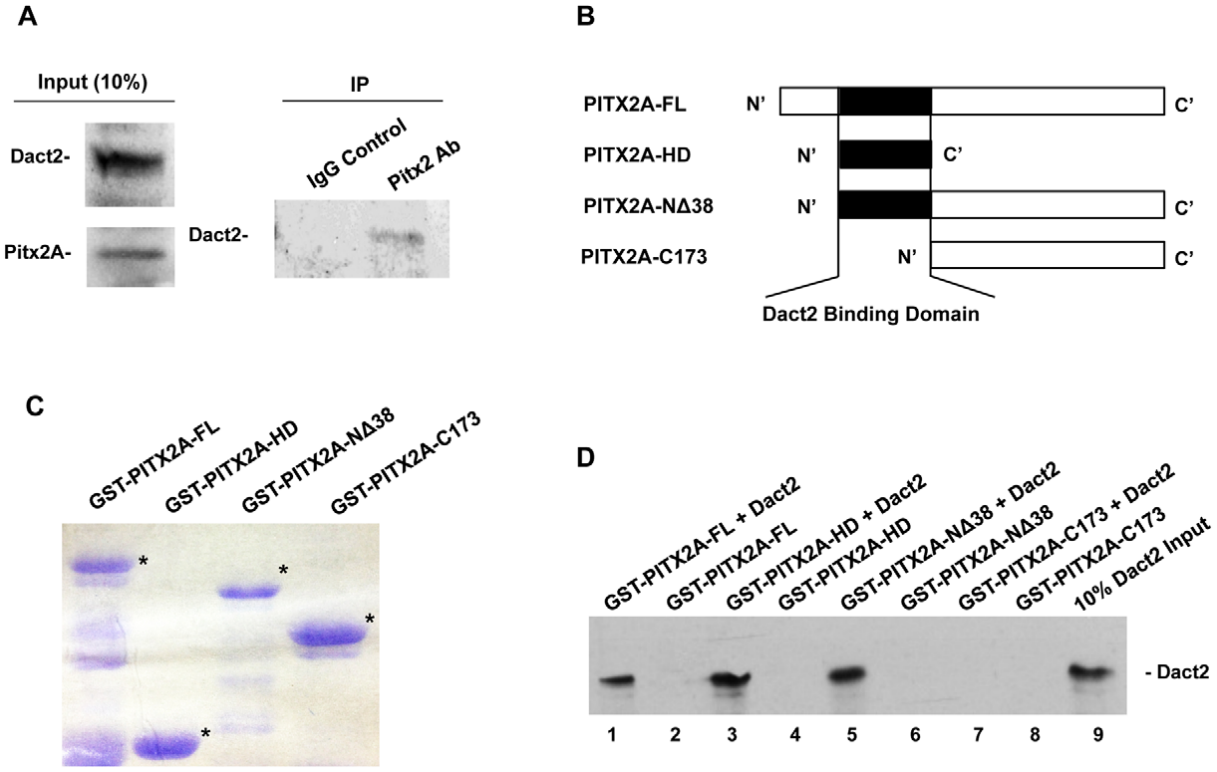
Dact2 and Pitx2 physically interact. (**A**) LS-8 cells were used for endogenous immunoprecipitation assays. Western Blots shows the high expression levels of Dact2 and Pitx2A in the 10 times diluted input LS-8 cell lysate. The right lane of the IP Western Blot shows Dact2 protein pulled down by Pitx2 antibody. Left lane shows the parallel IP performed using normal rabbit IgG as negative control. (**B**) Schematics of the gene structures of PITX2A truncations used in GST pull down assay. (**C**) Coomassie blue staining of the purified PITX2A truncated proteins fused with GST tag (bands with the correct sizes marked by *). (**D**) Dact2 Western blot of the GST pull-down assay. Dact2 pure protein was incubated with different truncated PITX2A in lane 1, 3, 5 and 7. Incubation of corresponding truncated PITX2A only controls were in lane 2, 4, 6 and 8. Lane 9 contains 10% Dact2 pure protein input. Results indicated Dact2 binds PITX2A through the homeodomain.

LS-8 cells express both Dact2 and Pitx2 and we used LS-8 cells to conduct endogenous immunoprecipitation assays. We were able to successfully pull down Dact2 proteins in the whole cell lysate by using a PITX2 antibody, compared to the IgG mock negative control (Fig. 7A).

A GST-pull down assay was used to confirm the PITX2-Dact2 interaction. Dact2 protein and immobilized GST-PITX2A fusion proteins were expressed in bacteria purified and used in the pull-down assays. Schematics showed the gene structures of PITX2A truncations (Fig. 7B). A coomassie blue staining of the purified GST-PITX2A proteins is shown (bands with the correct sizes marked by asterisk) (Fig. 7C). Dact2 bound to immobilized GST-PITX2-Full length, GST-PITX2-homeodomain, GST-PITX2- NΔ38 but not GST-PITX2- C173. Protein incubations without Dact2 were done as negative controls (Fig. 7D). This pull-down result suggests that Dact2 binds PITX2A through the homeodomain. Collectively, the above evidence demonstrates that Dact2 and Pitx2 co-localize in the cell, and physically interact under physiological conditions and in vitro. Pitx2, Lef-1 and β-catenin are associated as a complex when activating downstream gene expression [6]. A recent study employed IP assays to prove that Dact2 protein interacts with β-catenin and Lef-1 [22]. These data demonstrate that Dact2 is a part of the Pitx2- Lef-1- β-catenin complex and that Dact2 represses PITX2 transcriptional activity through a direct physical interaction.

### Dact2 knock down increases endogenous gene expression

**Figure 8:**
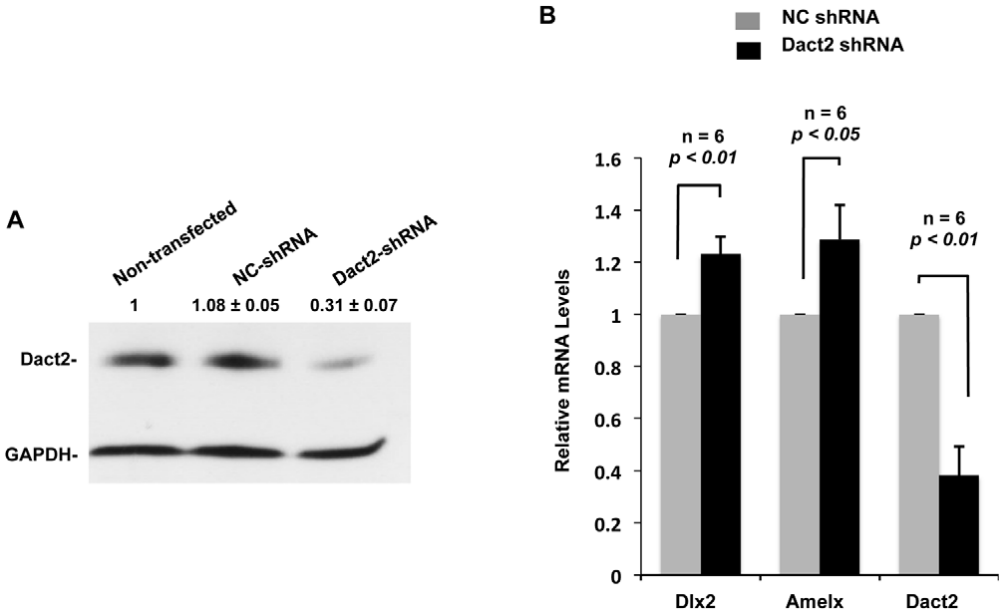
Knock down of *Dact2* activates endogenous Pitx2 target genes. (**A**) Knocking down of endogenous Dact2 in LS-8 cells by shRNA was shown by Western Blot. Negative control shRNA transfected cells show no change. GAPDH was probed as loading controls. Protein band intensities were quantified and shown as relative value ±SEM. (**B**) mRNAs were extracted from LS-8 cells transfected with *Dact2* shRNA or NC shRNA, and subjected to RT-PCRs and real-time PCRs. Relative expression levels of *Dlx2* and *Amelx* were analyzed and correlated with *Dact2* expression level. All Real-time PCRs were performed in triplicates and repeated six times.

To further investigate the effect of Dact2 on repressing the transcriptional hierarchy during tooth development, we generated shRNA expression plasmids to specifically knock down endogenous *Dact2* in dental epithelial cells and evaluate gene expression. The shRNA expression plasmids were transfected in LS-8 cells and Dact2 protein detected by Western blot. A decreased level of endogenous Dact2 was seen compared to the mock shRNA transfected cells (Fig. 8A). As we expected, higher *Dlx2* and *Amelx* mRNA levels were observed with decreased Dact2 (Fig. 8B). As a control the shRNA to Dact2 inhibited *Dact2* expression approximately 60% compared to controls (Fig. 8B). The 20% decrease in gene regulation seen in *Dlx2* and *Amelx* could potentially be higher if Dact2 was completely absence. These data suggest that Dact2 acts as a differentiation repressor in tooth epithelia by regulating Dlx2 and Amelx expression through a Wnt/β-catenin/Pitx2 pathway.

### Dact2 represses cell proliferation

**Figure 9:**
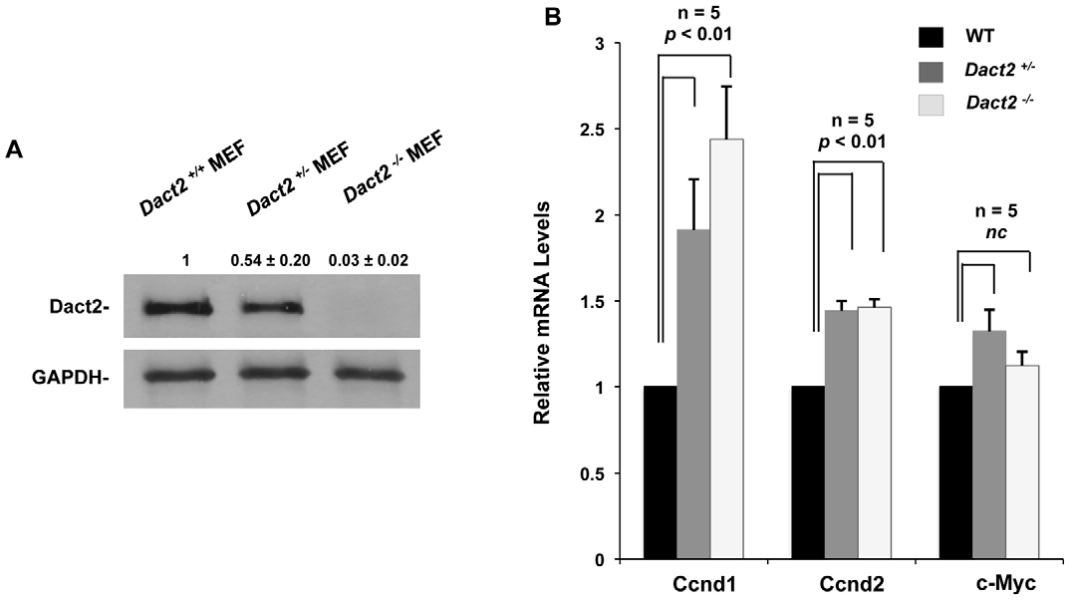
Dact2 down-regulates Wnt responsive proliferation markers. (**A**) MEF cells from *Dact2^−/−^, Dact2^+/−^ and Dact2^+/+^* embryos were lysed and analyzed by Western blots. GAPDH was probed as loading controls. Protein band intensities were quantified and shown as relative value ±SEM. (**B**) mRNAs extracted from MEF cells were subjected to RT-PCRs and real-time PCRs. Specific primers for proliferation markers *Ccnd1, Ccnd2* and *c-Myc* were used in the real-time PCRs to evaluate the relative expression level of these proliferation markers. All real-time PCRs were performed in triplicates and repeated five times.

We analyzed the *Dact2* null mice and only subtle phenotypes were observed, which was consistent with a previous study [13]. We isolated MEF cells from E13.5 littermate embryos with genotypes of *Dact2^−/−^, Dact2^+/−^* and wild type. Dact2 protein was detected by Western blot in wild type MEFs and both the *Dact2^+/−^* and *Dact2^−/−^* isolated MEFs showed reduced or undetectable Dact2 expression, respectively (Fig. 9A). Cyclin-D1 (Ccnd1), Cyclin-D2 (Ccnd2) and c-Myc are direct downstream target genes of the Wnt/β-catenin pathway [33], [34], [35], [36]. We evaluated these genes in MEFs by real-time PCR, and their mRNA levels were significantly down regulated, with the exception of c-Myc (Fig. 9B). As shown before, Cyclin-D1 and Cyclin-D2 were identified as downstream genes of Pitx2 [36]. Thus, activation of cyclin-D1 and cyclin-D2 by Pitx2 were subject to the inhibition by Dact2.

**Figure 10:**
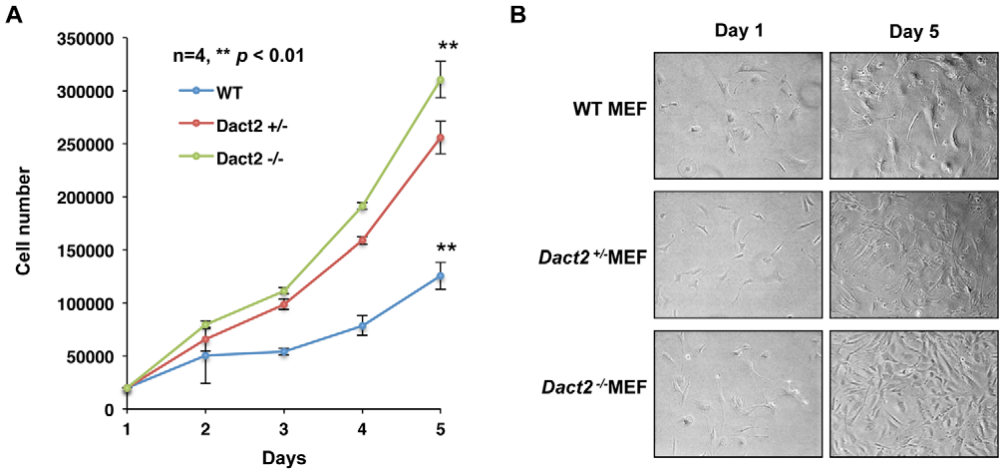
Dact2 represses cell proliferation. (**A**) 96-hour cell proliferation assays were performed with *Dact2^−/−^, Dact2^+/−^* and *Dact2^+/+^* MEF cells at passage 3. All cell counting were performed in triplicate. (**B**) Microscopic photos of seeded MEF cells at the beginning and end of the proliferation assay.

A series of cell proliferation assays were performed and the proliferation rates of MEF cells were negatively correlated with *Dact2* expression levels. The deletion of *Dact2* in MEF cells significantly increased the proliferation rates of these cells compared to wild type MEF cells (Fig. 10A). The cells plated on culture dishes and grown for 1 and 5 days are shown (Fig. 10B). These data indicated that Dact2 serves as a repressor of cell proliferation.

### Dact2 regulates β-catenin localization in the dental epithelium

**Figure 11:**
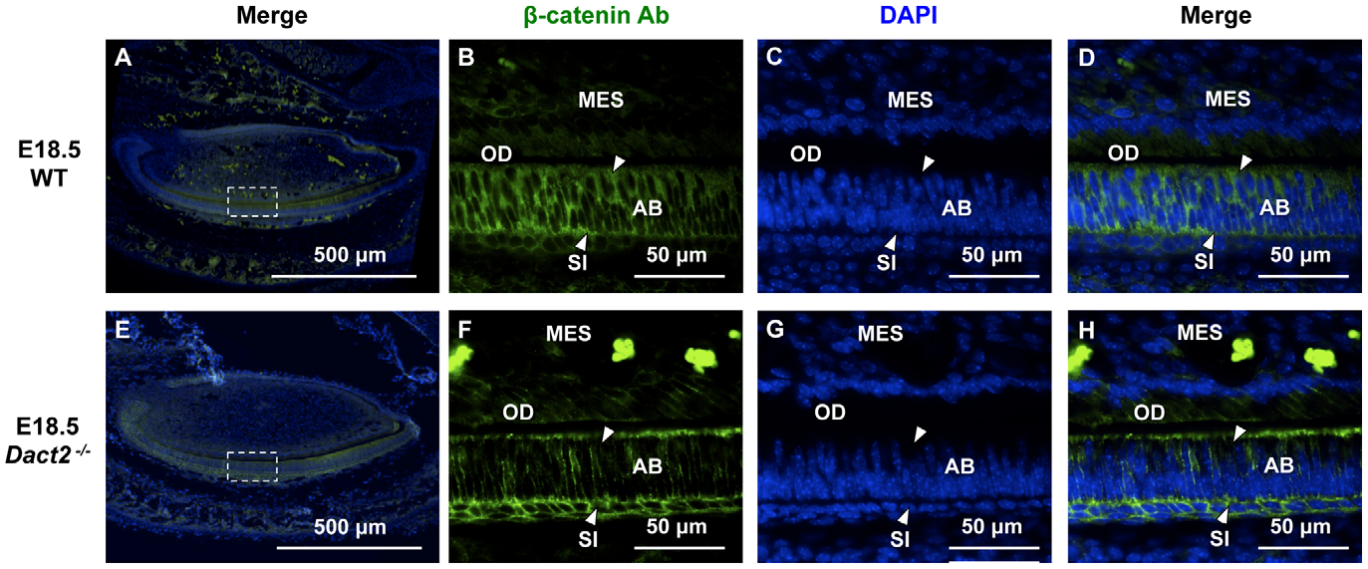
β-catenin cellular localization is changed in the dental epithelium of *Dact2* null mice. (**A** and **E**) E18.5 WT and *Dact2^−/−^* lower incisors were examined by immunohistochemistry for β-catenin expression. Boxed region were examined under higher magnification. (**B** and **F**) Detailed views of the labial dental epithelium were shown. β-catenin was labeled with FITC. (**C** and **G**) Nuclei were stained with DAPI. (**D** and **H**) Merged signals of FITC and DAPI are shown. Arrowheads indicate the differentially localized β-catenin. MES, mesenchyme; OD, odontoblasts; AB, ameloblast; SI, stratum intermedium.

Analyses of β-catenin localization in E18.5 wild type and *Dact2^−/−^* tooth germs shows β-catenin expression throughout the lower incisor dental epithelium from the cervical loops (Cl) (stem cell niche) to the differentiated ameloblast (Am) cells at the apical tip of the growing incisor (Fig. 11A, E). Interestingly, β-catenin nuclear localization is reduced in the *Dact2* null differentiated ameloblast (Am) cells (Fig. 11F) compared to wild type (Fig. 11B). β-catenin is redistributed to the apical and basal regions of the polarized ameloblasts cells deficient in Dact2. β-catenin is concentrated at the apical and basal ends of the polarized ameloblasts and increased in the stratum intermedium (SI) in the *Dact2* null E18.5 incisors (Fig. 11F). These data suggest that Dact2 regulates β-catenin cytoplasmic and nuclear distribution in differentiated ameloblast cells. Interestingly, the levels of β-catenin are unchanged in the cervical loops of the *Dact2* null mice compared to wild type embryos (data not shown).

## Discussion

The Dact proteins appear to stabilize β-catenin through unknown mechanisms and can act as inhibitors or activators of Wnt/β-catenin signaling [7], [37], [38]. In Zebrafish Dact2 (Dpr2) is expressed on the dorsal side of the embryo and is required for morphogenetic movements during gastrulation [10], [11]. Dact2 (Dpr2) expression in Zebrafish is regulated by β-catenin signaling and inhibits Nodal signaling during morphogenesis [10], [11]. *Xenopus* Dpr binds to Dsh and stabilizes the β-catenin degradation pathway, which results in the degradation of soluble β-catenin. The loss of soluble β-catenin inhibits the activation of downstream β-catenin dependent target genes [7]. The inhibition of Dpr activates β-catenin responsive genes [7]. *Xenopus* Dpr phosphorylation promotes Wnt/β-catenin signaling activity and the unphosphorylated form inhibits Wnt/β-catenin activity [39]. Thus, phosphorylation of Dact proteins regulates their activity and Wnt/β-catenin signaling. Furthermore, mammalian Dact1 inhibits the binding of Lef-1 with β-catenin and promotes the interaction of Lef-1 with histone deacetylase (HDAC1) a co-repressor [9], [38].

*Dact2* null mice have been previously reported and similar to our analyses the embryos developed normally and grew to adulthood without obvious phenotypic defects [13]. Dact2 was shown to inhibit TGFβ signaling in both mice and zebrafish [13], [40]. The *Dact2* null mice revealed a specific effect in the epidermal keratinocytes and a role in wound healing [13]. Dact2 appears to inhibit re-epithelialization by modulating TGFβ signaling [13]. Our study provides new mechanisms for Dact2 through its interaction with the Pitx2 transcription factor.

### Dact2 expression and regulation by Pitx2

*Dact2* expression has been shown during tooth development and *Dact2* transcripts are restricted to the dental epithelium [8], [23]. *Dact2* expression was observed throughout tooth development in the dental epithelium from E11.5 to P2. *Dact2* expression was detected in cells of the epithelial enamel organ including the stellate reticulum, stratum intermedium and cervical loop (stem cell niche) as well as the inner and outer dental epithelium [23]. Interestingly, *Dact2* transcripts are downregulated in differentiated ameloblasts and completely lost by P8 in the tooth germ [23]. However, the mechanism of Dact2 during tooth development was not addressed and in this report we reveal new molecular mechanisms for Dact2 during tooth morphogenesis.

The transcriptional regulation of Dact2 was reported to be through a Wnt/β-catenin pathway but the direct activation was unknown. We demonstrate that Pitx2 directly regulates endogenous Dact2 expression. Pitx2 interacts with many transcription factors to regulate gene expression [3], [5]. Dact2 represses Pitx2 transcriptional activation of genes involved in both early (Dlx2 and FoxJ1) and late (amelogenin) tooth development. We show that this repression is independent of the interaction of Pitx2 with β-catenin. Thus, Dact2 expression is activated by Pitx2 and feeds back to repress Pitx2 transcriptional activity. It was previously shown that Dact2 inhibits Lef-1 interaction with β-catenin, however, Dact2 does not appear to inhibit the interaction of Pitx2 with β-catenin or Lef-1, but modulates their synergistic transcriptional activities.

### Dact2 interacts with Pitx2

Dact2 is both nuclear and cytoplasm localized in the cell and colocalizes with Pitx2 in the nucleus. Dact2 binds to Pitx2 directly and knockdown of Dact2 expression results in an increase in Pitx2 target gene expression. Our data demonstrate that Dact2 regulates gene expression during tooth development by a direct interaction with Pitx2. This regulation of gene expression appears to be linked with Wnt/β-catenin signaling through β-catenin interactions with Lef-1 and Pitx2. From our studies and previous reports it is clear that Dact2 is interacting directly with transcription factors as well as regulating the function of soluble β-catenin in the cell.

### Dact2 regulates cell proliferation and β-catenin localization

We demonstrate that Dact2 represses cell proliferation and these data are similar to the report of decreased Dact2 expression causing a re-epithelialization during wound healing [13]. These cell processes are linked to β-catenin and cyclin D1 and D2 expression and we show that Dact2 represses both cyclin D1 and D2. Analyses of β-catenin expression and cellular localization during lower incisor development revealed a redistribution of β-catenin in *Dact2* null differentiated ameloblast cells. In the differentiated ameloblast cells Dact2 allows for β-catenin accumulation in the nuclei and membrane components. Without Dact2, β-catenin is localized to the apical and basal ends of the ameloblasts as well as the stratum intermedium. Dact2 appears to be required for the nuclear localization of β-catenin in these differentiated cells to allow for its interaction with Pitx2 and Lef-1 transcription factors. This would be required to activate gene expression during late tooth morphogenesis.

The model we proposed contains a negative auto-regulation loop, in which Dact2 represses the activation of its own promoter through inhibiting Pitx2 transcriptional activity. Since both Pitx2 and Dact2 are highly conserved between mouse and zebrafish, this mechanism provides an explanation for the morpholino knockdown experiments conducted in zebrafish resulting a possible negative auto-regulation of zebrafish Dact2 [11].

**Figure 12:**
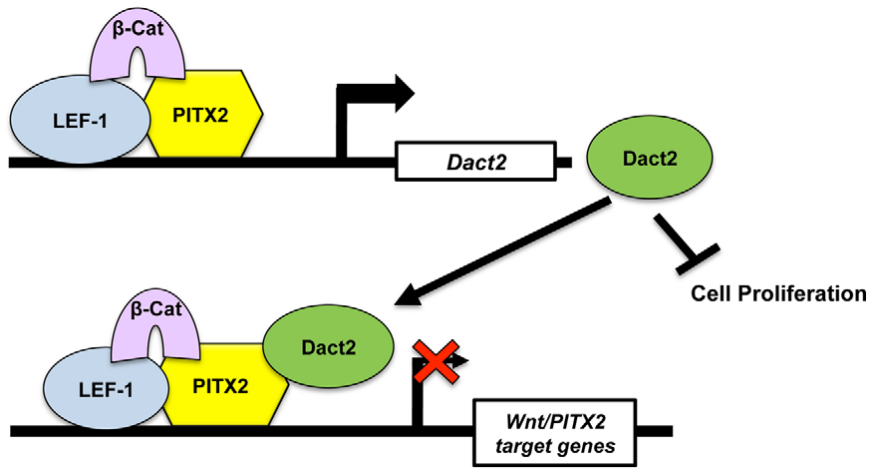
Model for the mechanism of Dact2. *Dact2* is a direct downstream target gene of Pitx2 and Wnt signaling. Dact2 negatively feeds back and represses the transcriptional activity of Pitx2, and in turn inhibits Wnt/β-catenin signaling responsive proliferation. Dact2 also inhibits Wnt signaling responsive cell proliferation.

It was well established that excessive Wnt signaling leads to supernumery teeth formation, while insufficient Wnt signaling leads to arrest of early tooth development [20], [41], [42], [43], [44]. Without the inhibition of Dact2 on Wnt/β-catenin signaling in *Dact2^−/−^* mouse, a supernumery teeth phenotype was expected. Although consistent with the previous study [13], it is surprising that *Dact2^−/−^* mice don't exhibit any significant phenotype in the craniofacial region. This may be due to the functional redundancy within Dact paralogs, especially when Dact1 and Dact2 are both highly expressed in the dental epithelium. Although Dact1 and Dact2 both are able to inhibit Wnt/β-catenin signaling, the direct targets of inhibitor interaction are divergent. Dvl proteins are targeted by Dact1 and reside in the Wnt/β-catenin pathway at a higher hierarchy, which means the regulation of Wnt target gene expression is in a macro scale. However, Pitx2 is targeted by Dact2 and acts as an effector downstream of the Wnt/β-catenin pathway, which means Dact2 regulates a narrower spectrum of Wnt target genes in a more precise manner (Fig. 12). These data indicate Dact2 plays a role in Wnt signaling regulation as a “fine tune” mechanism, while Dact1 serves an “on and off” switch. This is a possible reason for the lack of a severe developmental phenotype in *Dact2^−/−^* mice.

## Supporting Information

Figure S1 ***Dact2*** **expressed in dental and oral epithelia.** (**A**) Microarray analyses of epithelial and mesenchyme compartments of P1 mouse incisors. Shown is a heat map of mRNA expression. *Dact2* gene is highly expressed in the tooth epithelium. Known epithelial and mesenchymal markers are shown in the bottom as controls. (**B**) Western blots showed that endogenous Dact2 protein highly expresses in the LS-8 cells, which is an oral epithelial cell line. In contrast, no detectable expression was seen in the MDPC cells, an odontoblast-like cell line. (**C**). Real-time PCRs were performed with LS-8 cells and MDPC cells to show the relative expression of three *Dact* family genes. Expression levels of *Dact* genes were normalized to β-actin across two cell lines. All real-time PCRs were performed in triplicates and repeated at five times.
(TIF)

Figure S2 **Dact2 expression pattern overlaps with Pitx2 during tooth development.** (**A**) LacZ staining with eosin counter staining on E14.5 *Pitx2 ^cre/+^ Rosa26^+/−^* mice lower incisor germ. (**B**) Immunohistochemistry showed endogenous Dact2 protein stained by FITC conjugated antibody in E14.5 lower incisor germ. Nuclei were stained by DAPI. White dotted lines indicate the mesenchyme-epithelium boundaries of incisor germs. Scale bar represents 100 μm.
(TIF)

Figure S3 **Nonconserved Pitx2 binding motif in the Dact2 promoter does not bind Pitx2.** (**A**) Schematic of the location of the nonconserved binding site on *Dact2* 10 kb promoter at −3719 bp indicated by a white arrowhead. The location of the sense primer (−3753 bp) and the antisense primer (−3517 bp) are shown for amplification of the immunoprecipitated chromatin. (**B**) DNA from endogenous ChIP assay performed in Figure 2 was used to evaluate the enrichment of this nonconserved binding motif. Lane 5 contains the PCR marker. Lane 1 shows the *Dact2* primers-only control. Lane 2 is the amplified fragment from immunoprecipitation using normal rabbit immunoglobulin G. Lane 3 is Pitx2 antibody immunoprecipitated chromatin amplified using the specific *Dact2* promoter primers. Lane 4 is the chromatin input amplified using the Dact2 primers. Missing band in lane 3 indicate this putative binding site is not functional. All PCR products were sequenced to confirm their identity.
(TIF)

Figure S4 **Dact2 shRNA rescues Dact2 inhibition of the TOPFlash reporter.** (**A**) Combinations of *CMV-Lef1, CMV-PITX2A, CMV-Dact2* and Dact2 shRNA were co-transfected in CHO cells with TOPflash reporter. ++ indicate double dosage of transfected Dact2 shRNA plasmid. (**B**) FOPflash reporter were transfected instead of TOPflash with combinations of *CMV-Lef1, CMV-Dact2* and Dact2 shRNA. No shRNA rescuing effect was seen in the results, indicating FOPflash activation was not specific due to Dact2 overexpression. All luciferase activities are shown as mean-fold activation compared with the reporter plasmid co-transfected with empty CMV expression plasmid (± SEM from five independent experiments).
(TIF)

Figure S5 **shRNA knocks down Dact2 protein in LS-8 cells.** (**A**) Dact2 protein was probed by Dact2 primary antibody and labeled with FITC in non-transfected LS-8 cells. (**B**) Similar level of Dact2 protein staining was seen in NC-shRNA transfected LS-8 cells. (**C**) Dact2 protein staining was significantly lower in Dact2 shRNA transfected LS-8 cells. Results support efficiency of Dact2 shRNA, as well as the specificity of Dact2 antibody in immunocytostaining experiments shown in Fig. 1 and Fig. 6. All cells were counter staining with DAPI to show nuclei. Scale bars represent 50 μm.
(TIF)

## References

[1] Pispa J, Thesleff I (2003) Mechanisms of ectodermal organogenesis. Dev Biol 262: 195–205. 14550785 10.1016/s0012-1606(03)00325-7

[2] Tucker A, Sharpe P (2004) The cutting-edge of mammalian development; how the embryo makes teeth. Nat Rev Genet 5: 499–508. 15211352 10.1038/nrg1380

[3] Venugopalan SR, Li X, Amen MA, Florez S, Gutierrez D, et al (2011) Hierarchical interactions of homeodomain and forkhead transcription factors in regulating odontogenic gene expression. J Biol Chem 286: 21372–21383. 21504905 10.1074/jbc.M111.252031 PMC3122197

[4] Green PD, Hjalt TA, Kirk DE, Sutherland LB, Thomas BL, et al (2001) Antagonistic Regulation of Dlx2 Expression by PITX2 and Msx2: Implications for Tooth Development. Gene Expr 9: 265–281. 11763998 10.3727/000000001783992515 PMC5964948

[5] Vadlamudi U, Espinoza HM, Ganga M, Martin DM, Liu X, et al (2005) PITX2, β-catenin, and LEF-1 Interact to Synergistically Regulate the LEF-1 promoter. J Cell Sci 118: 1129–1137. 15728254 10.1242/jcs.01706

[6] Amen M, Liu X, Vadlamudi U, Elizondo G, Diamond E, et al (2007) PITX2 and β-catenin Interactions Regulate Lef-1 Isoform Expression. Mol Cell Biol 27: 7560–7573. 17785445 10.1128/MCB.00315-07 PMC2169058

[7] Cheyette BNR, Waxman JS, Miller JR, Takemaru K-I, Sheldahl LC, et al (2002) Dapper, a Dishevelled-Associated Antagonist of beta-Catenin and JNK Signaling, Is Required ofr Notochord Formation. Dev Cell 2: 449–461. 11970895 10.1016/s1534-5807(02)00140-5

[8] Fisher DA, Kivimae S, Hoshino J, Suriben R, Martin P-M, et al (2006) Three Dact Gene Family members Are Expressed During Embryonic Development and in the Adult Brains of Mice. Dev Dyn 235: 2620–2630. 16881060 10.1002/dvdy.20917

[9] Zhang L, Gao X, Wen J, Ning Y, Chen Y-G (2006) Dapper 1 antagonizes Wnt signaling by promoting dishevelled degradation. J Biol Chem 281: 8607–8612. 16446366 10.1074/jbc.M600274200

[10] Waxman JS (2004) Zebrafish Dapper1 and Dapper2 play distinct roles in Wnt-mediated developmental processes. Development. 5909–5921. 10.1242/dev.01520 15539487

[11] Zhang L (2004) Zebrafish Dpr2 Inhibits Mesoderm Induction by Promoting Degradation of Nodal Receptors. Science 306: 114–117. 15459392 10.1126/science.1100569

[12] Suriben R, Kivimäe S, Fisher DAC, Moon RT, Cheyette BNR (2009) Posterior malformations in Dact1 mutant mice arise through misregulated Vangl2 at the primitive streak. Nat Genet 41: 977–985. 19701191 10.1038/ng.435 PMC2733921

[13] Meng F, Cheng X, Yang L, Hou N, Yang X, et al (2008) Accelerated re-epithelialization in Dpr2-deficient mice is associated with enhanced response to TGFβ signaling. J Cell Sci 121: 2904–2912. 18716284 10.1242/jcs.032417

[14] Lee W-C, Hough MT, Liu W, Ekiert R, Lindström NO, et al (2010) Dact2 is expressed in the developing ureteric bud/collecting duct system of the kidney and controls morphogenetic behavior of collecting duct cells. Am J Physiol Renal Physiol 299: F740–751. 20685821 10.1152/ajprenal.00148.2010

[15] Cadigan KM, Nusse R (1997) Wnt signaling: a common theme in animal development. Genes Dev 11: 3286–3305. 9407023 10.1101/gad.11.24.3286

[16] Nelson WJ (2004) Convergence of Wnt, β-Catenin, and Cadherin Pathways. Science 303: 1483–1487. 15001769 10.1126/science.1094291 PMC3372896

[17] Wehrli M, Dougan ST, Caldwell K, O'Keefe L, Schwartz S, et al (2000) arrow encodes an LDL-receptor-related protein essential for Wingless signalling. Nature 407: 527–530. 11029006 10.1038/35035110

[18] Amendt BA, Sutherland LB, Russo AF (1999) Multifunctional Role of the Pitx2 Homeodomain Protein C-Terminal Tail. Mol Cel Biol 19: 7001–7010. 10.1128/mcb.19.10.7001 PMC84695 10490637

[19] Logan CY, Nusse R (2004) The Wnt signaling pathway in development and disease. Annu Rev Cell Dev Biol 20: 781–810. 15473860 10.1146/annurev.cellbio.20.010403.113126

[20] Liu F, Chu EY, Watt B, Zhang Y, Gallant NM, et al (2008) Wnt/beta-catenin signaling directs multiple stages of tooth morphogenesis. Dev Biol 313: 210–224. 18022614 10.1016/j.ydbio.2007.10.016 PMC2843623

[21] Chen J, Lan Y, Baek J-A, Gao Y, Jiang R (2009) Wnt/beta-catenin signaling plays an essential role in activation of odontogenic mesenchyme during early tooth development. Dev Biol 334: 174–185. 19631205 10.1016/j.ydbio.2009.07.015 PMC2752344

[22] Kivimäe S, Yang XY, Cheyette BNR (2011) All Dact (Dapper/Frodo) scaffold proteins dimerize and exhibit conserved interactions with Vangl, Dvl, and serine/threonine kinases. BMC Biochemistry 12: 33. 21718540 10.1186/1471-2091-12-33 PMC3141656

[23] Kettunen P, Kivimäe S, Keshari P, Klein OD, Cheyette BNR, et al (2010) Dact1–3 mRNAs exhibit distinct expression domains during tooth development. Gene Expr Patterns 10: 140–143. 20170752 10.1016/j.gep.2010.02.002 PMC2849867

[24] Amendt BA, Sutherland LB, Semina E, Russo AF (1998) The Molecular Basis of Rieger Syndrome: Analysis of Pitx2 Homeodomain Protein Activities. J Biol Chem 273: 20066–20072. 9685346 10.1074/jbc.273.32.20066

[25] Cox CJ, Espinoza HM, McWilliams B, Chappell K, Morton L, et al (2002) Differential Regulation of Gene Expression by PITX2 Isoforms. J Biol Chem 277: 25001–25010. 11948188 10.1074/jbc.M201737200

[26] Chen LS, Couwenhoven RI, Hsu D, Luo W, Snead ML (1992) Maintenance of Amelogenin Gene Expression by Transformed Epithelial Cells of Mouse Enamel Organ. Archs oral Biol 37: 771–778. 10.1016/0003-9969(92)90110-t 1444889

[27] Diamond E, Amen M, Hu Q, Espinoza HM, Amendt BA (2006) Functional Interactions Between Dlx2 and Lymphoid Enhancer Factor Regulate Msx2. Nuc Acids Res 34: 5951–5965. 10.1093/nar/gkl689 PMC1635299 17068080

[28] Ovcharenko I, Nobrega MA, Loots GG, Stubbs L (2004) ECR Browser: a tool for visualizing and accessing data from comparisons of multiple vertebrate genomes. Nuc Acids Res 32: W280–286. 10.1093/nar/gkh355 PMC441493 15215395

[29] Zhang Z, Wlodarczyk BJ, Niederreither K, Venugopalan S, Florez S, et al (2011) Fuz Regulates Craniofacial Development Through Tissue Specific Responses to Signaling Factors. PLoS ONE 6: e24608. 21935430 10.1371/journal.pone.0024608 PMC3173472

[30] Hjalt TA, Semina EV, Amendt BA, Murray JC (2000) The Pitx2 Protein in Mouse Development. Dev Dyn 218: 195–200. 10822271 10.1002/(SICI)1097-0177(200005)218:1<195::AID-DVDY17>3.0.CO;2-C

[31] Cao H, Wang J, Li X, Florez S, Huang Z, et al (2010) MicroRNAs play a critical role in tooth development. J Dent Res 89: 779–784. 20505045 10.1177/0022034510369304 PMC3014323

[32] Gao X, Wen J, Zhang L, Li X, Ning Y, et al (2008) Dapper1 Is a Nucleocytoplasmic Shuttling Protein That Negatively Modulates Wnt Signaling in the Nucleus. J Biol Chem 283: 35679–35688. 18936100 10.1074/jbc.M804088200

[33] He T-C, Sparks AB, Rago C, Hermeking H, Zawel L, et al (1998) Identification of c-MYC as a Target of the APC Pathway. Science 281: 1509–1512. 9727977 10.1126/science.281.5382.1509

[34] Shtutman M, Zhurinsky J, Simcha I, Albanese C, D'Amico M, et al (1999) The cyclin D1 gene is a target of the beta-catenin/LEF-1 pathway. Proc Natl Acad Sci USA 96: 5522–5527. 10318916 10.1073/pnas.96.10.5522 PMC21892

[35] Tetsu O, McCormick F (1999) β-Catenin regulates expression of cyclin D1 in colon carcinoma cells. Nature 398: 422–426. 10201372 10.1038/18884

[36] Huang Y, Guigon CJ, Fan J, Cheng S-Y, Zhu G-Z (2010) Pituitary homeobox 2 (PITX2) promotes thyroid carcinogenesis by activation of cyclin D2. Cell Cycle 9. 10.4161/cc.9.7.11126 20372070

[37] Gloy J, Hikasa H, Sokol SY (2002) Frodo interacts with Dishevelled to transduce Wnt signals. Nat Cell Biol 4: 351–357. 11941372 10.1038/ncb784

[38] Hikasa H, Sokol SY (2004) The involvement of Frodo in TCF-dependent signaling and neural tissue development. Development 131: 4725–4734. 15329348 10.1242/dev.01369

[39] Teran E, Branscomb AD, Seeling JM (2009) Dpr Acts as a molecular switch, inhibiting Wnt signaling when unphosphorylated, but promoting Wnt signaling when phosphorylated by casein kinase Idelta/epsilon. PLoS ONE 4: e5522. 19440376 10.1371/journal.pone.0005522 PMC2679210

[40] Su Y, Zhang L, Gao X, Meng F, Wen J, et al (2007) The evolutionally conserved activity of Dapper2 in antagonizing TGF-beta signaling. FASEB J 21: 682–690. 17197390 10.1096/fj.06-6246com

[41] van Genderen C, Okamura RM, Farinas I, Quo R-G, Parslow TG, et al (1994) Development of several organs that require inductive epithelial-mesenchymal interactions is impared in LEF-1-deficient mice. Genes Dev 8: 2691–2703. 7958926 10.1101/gad.8.22.2691

[42] Andl T, Reddy ST, Gaddapara T, Millar SE (2002) WNT signals are required for the initiation of hair follicle development. Dev Cell 2: 643–653. 12015971 10.1016/s1534-5807(02)00167-3

[43] Järvinen E, Salazar-Ciudad I, Birchmeier W, Taketo MM, Jernvall J, et al (2006) Continuous tooth generation in mouse is induced by activated epithelial Wnt/beta-catenin signaling. Proc Natl Acad Sci USA 103: 18627–18632. 17121988 10.1073/pnas.0607289103 PMC1693713

[44] Wang X-P, O'Connell DJ, Lund JJ, Saadi I, Kuraguchi M, et al (2009) Apc inhibition of Wnt signaling regulates supernumerary tooth formation during embryogenesis and throughout adulthood. Development 136: 1939–1949. 19429790 10.1242/dev.033803 PMC2680115

