# Supplementary figures and images for: Dact2 Represses PITX2 Transcriptional Activation and Cell Proliferation through Wnt/beta-Catenin Signaling during Odontogenesis
Source: PLoS One. 2013 Jan 22;8(1):e54868. doi: 10.1371/journal.pone.0054868 (PMC3551926; doi:10.1371/journal.pone.0054868)

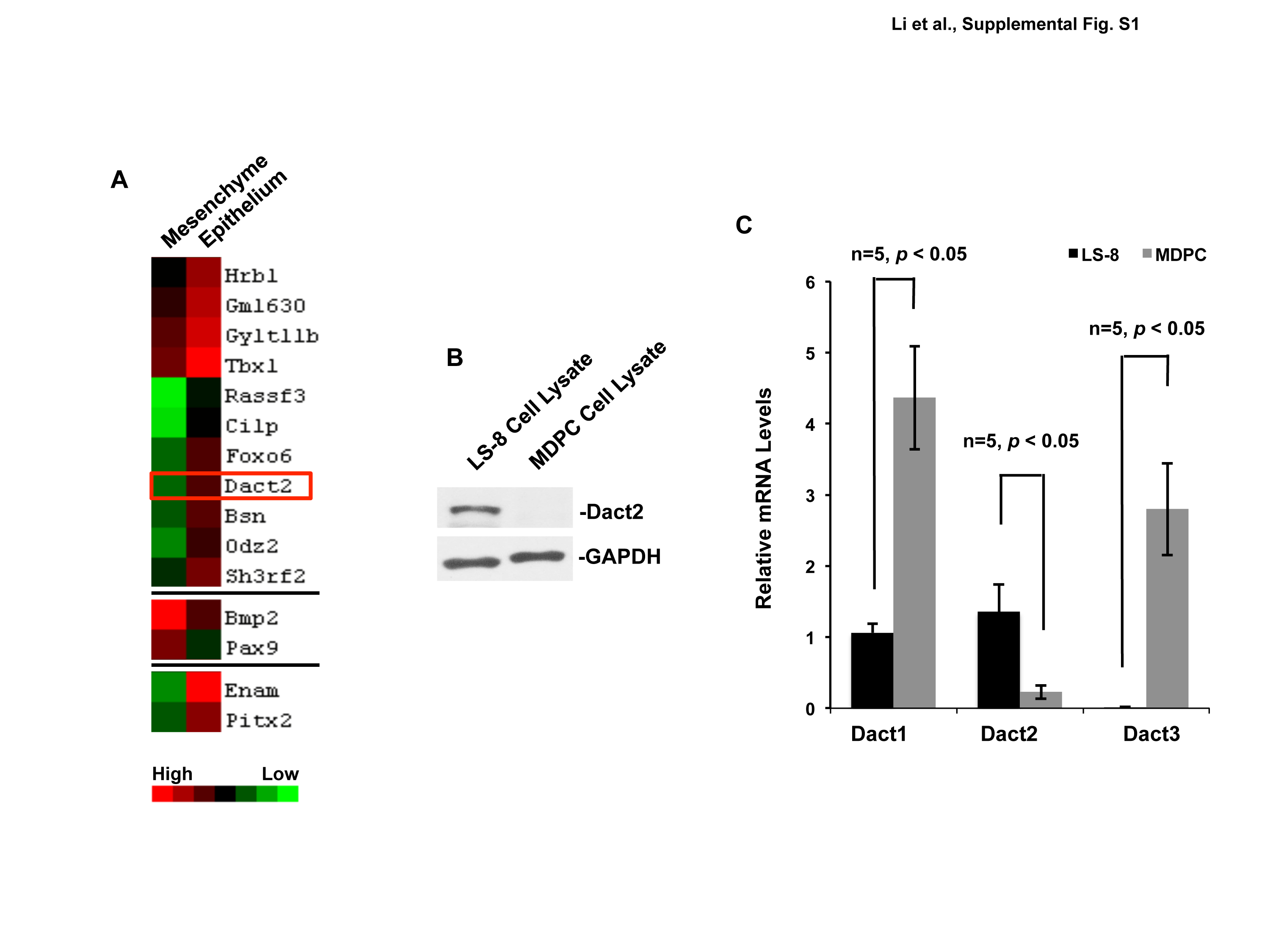

Supplement: Figure S1 — Dact2 expressed in dental and oral epithelia. (A) Microarray analyses of epithelial and mesenchyme compartments of P1 mouse incisors. Shown is a heat map of mRNA expression. Dact2 gene is highly expressed in the tooth epithelium. Known epithelial and mesenchymal markers are shown in the bottom as controls. (B) Western blots showed that endogenous Dact2 protein highly expresses in the LS-8 cells, which is an oral epithelial cell line. In contrast, no detectable expression was seen in the MDPC cells, an odontoblast-like cell line. (C). Real-time PCRs were performed with LS-8 cells and MDPC cells to show the relative expression of three Dact family genes. Expression levels of Dact genes were normalized to β-actin across two cell lines. All real-time PCRs were performed in triplicates and repeated at five times. (TIF) [file pone.0054868.s001.tif]

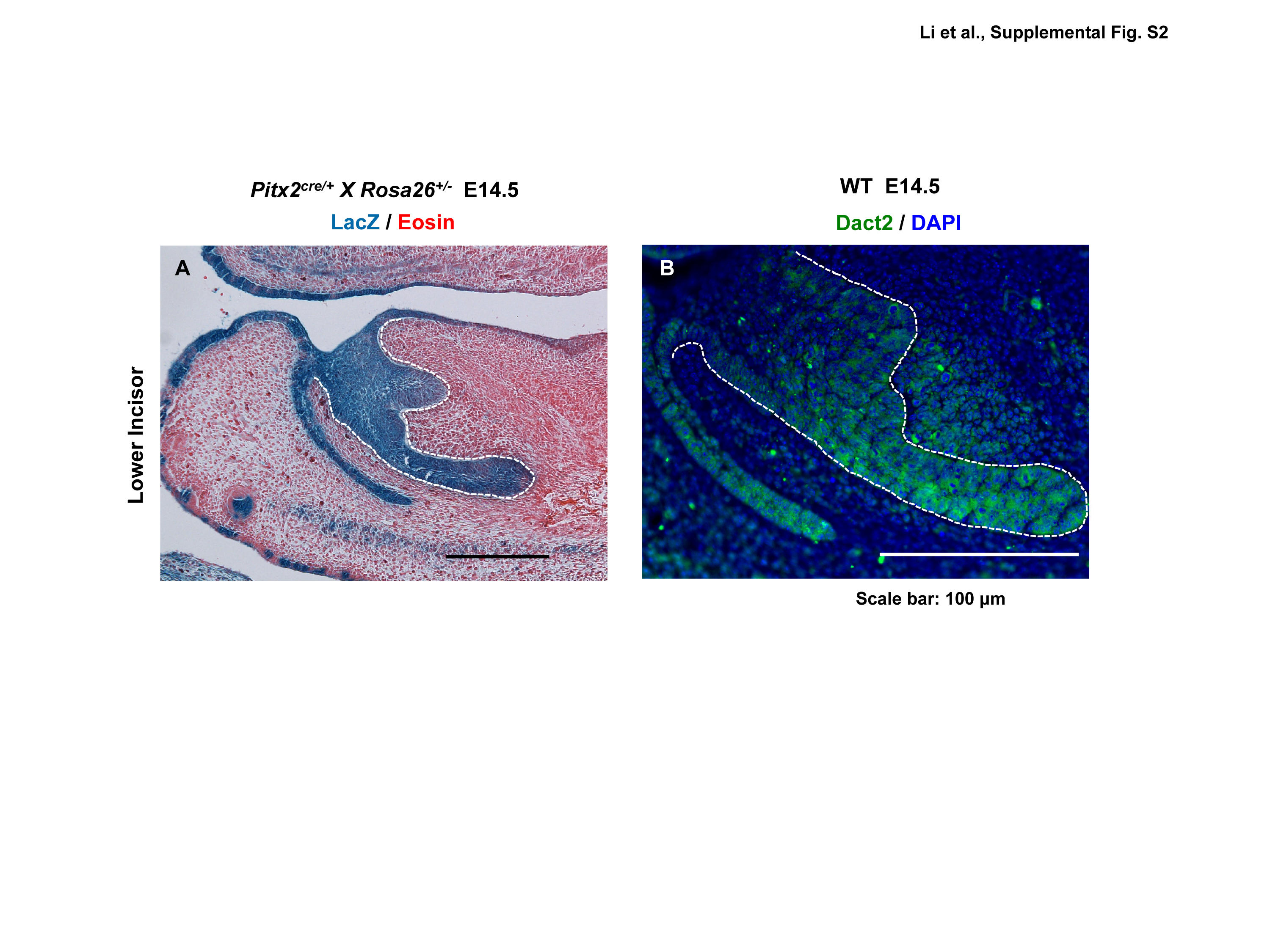

Supplement: Figure S2 — Dact2 expression pattern overlaps with Pitx2 during tooth development. (A) LacZ staining with eosin counter staining on E14.5 Pitx2 cre/+ Rosa26+/− mice lower incisor germ. (B) Immunohistochemistry showed endogenous Dact2 protein stained by FITC conjugated antibody in E14.5 lower incisor germ. Nuclei were stained by DAPI. White dotted lines indicate the mesenchyme-epithelium boundaries of incisor germs. Scale bar represents 100 μm. (TIF) [file pone.0054868.s002.tif]

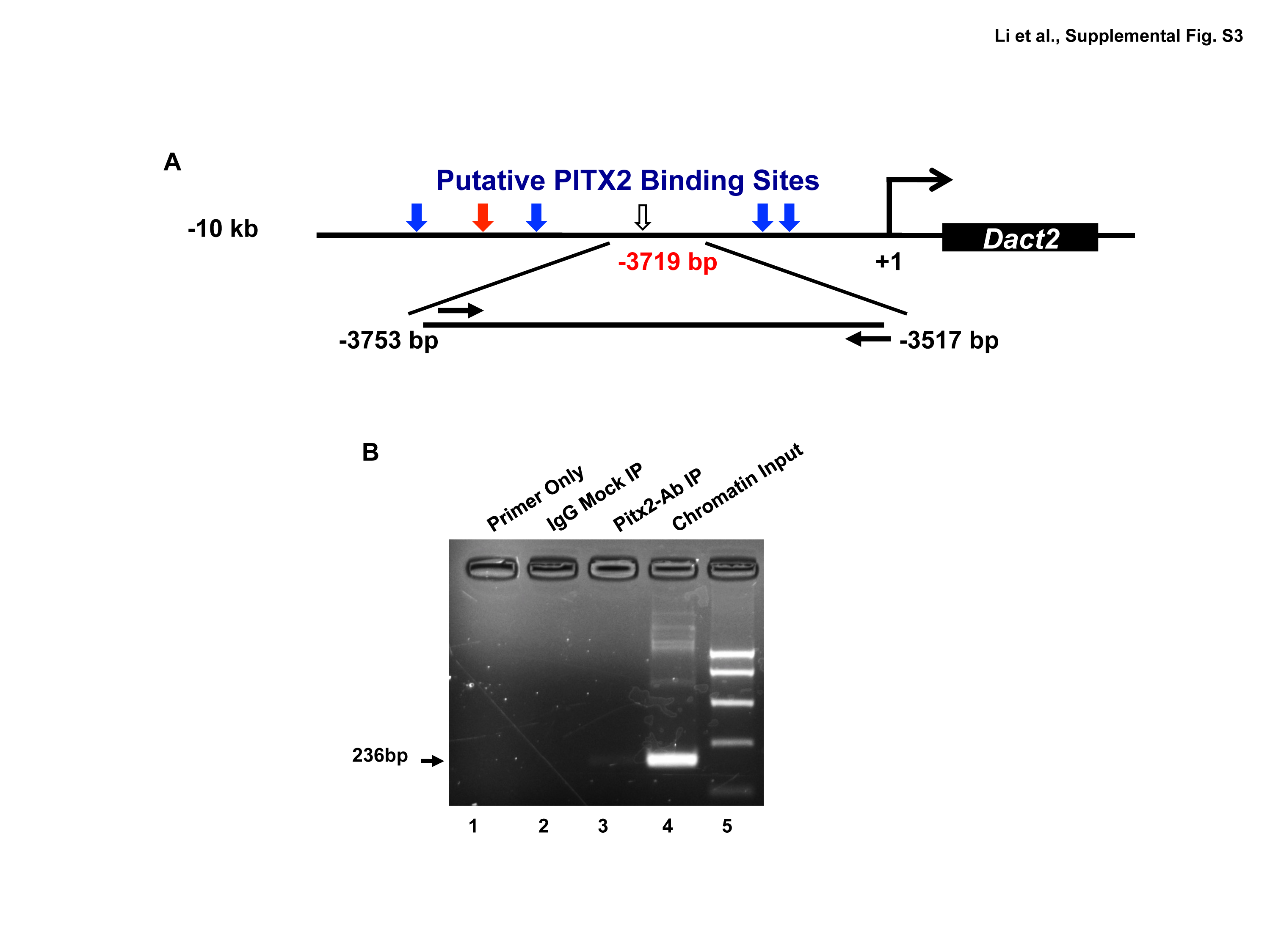

Supplement: Figure S3 — Nonconserved Pitx2 binding motif in the Dact2 promoter does not bind Pitx2. (A) Schematic of the location of the nonconserved binding site on Dact2 10 kb promoter at −3719 bp indicated by a white arrowhead. The location of the sense primer (−3753 bp) and the antisense primer (−3517 bp) are shown for amplification of the immunoprecipitated chromatin. (B) DNA from endogenous ChIP assay performed in Figure 2 was used to evaluate the enrichment of this nonconserved binding motif. Lane 5 contains the PCR marker. Lane 1 shows the Dact2 primers-only control. Lane 2 is the amplified fragment from immunoprecipitation using normal rabbit immunoglobulin G. Lane 3 is Pitx2 antibody immunoprecipitated chromatin amplified using the specific Dact2 promoter primers. Lane 4 is the chromatin input amplified using the Dact2 primers. Missing band in lane 3 indicate this putative binding site is not functional. All PCR products were sequenced to confirm their identity. (TIF) [file pone.0054868.s003.tif]

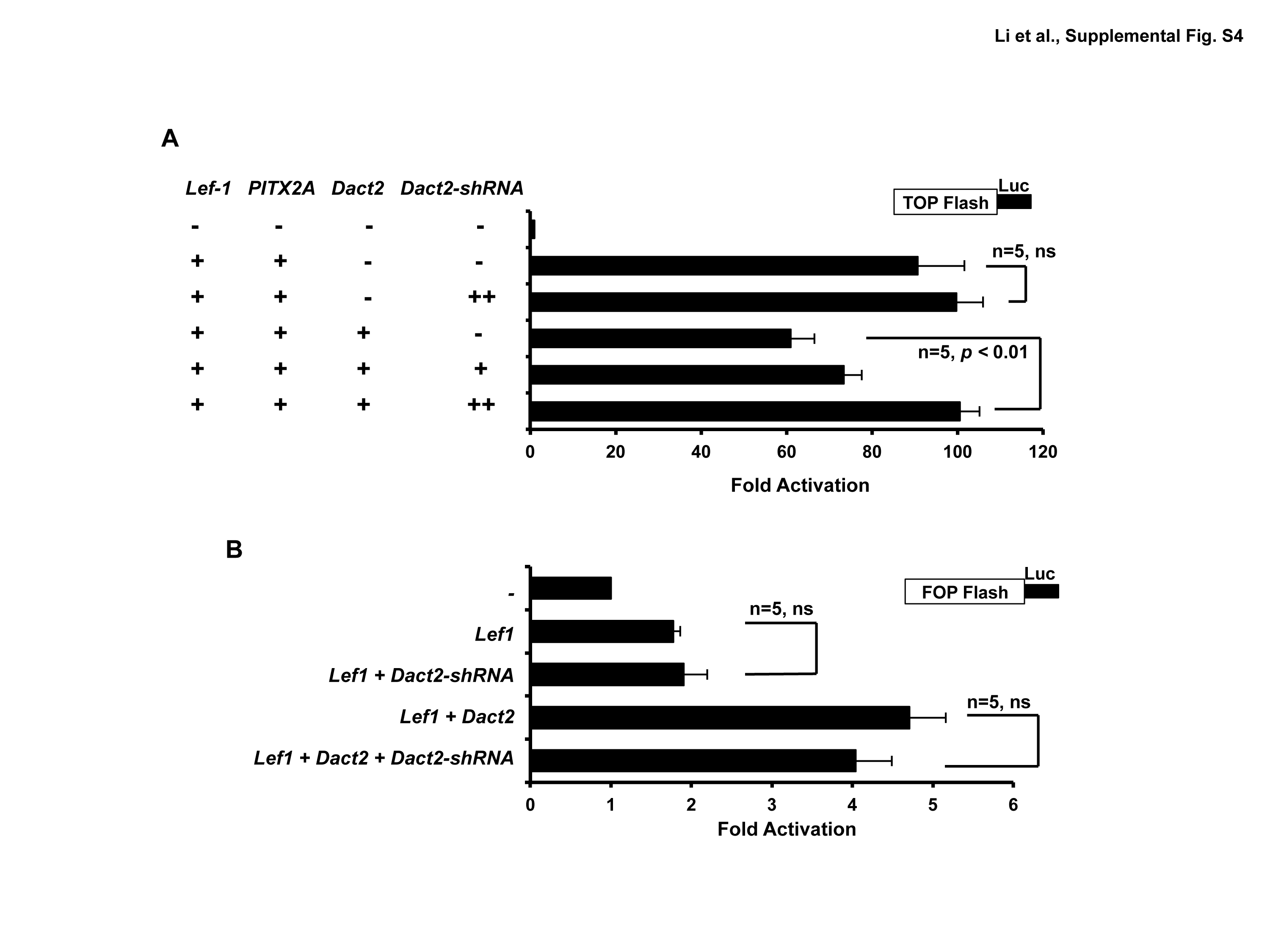

Supplement: Figure S4 — Dact2 shRNA rescues Dact2 inhibition of the TOPFlash reporter. (A) Combinations of CMV-Lef1, CMV-PITX2A, CMV-Dact2 and Dact2 shRNA were co-transfected in CHO cells with TOPflash reporter. ++ indicate double dosage of transfected Dact2 shRNA plasmid. (B) FOPflash reporter were transfected instead of TOPflash with combinations of CMV-Lef1, CMV-Dact2 and Dact2 shRNA. No shRNA rescuing effect was seen in the results, indicating FOPflash activation was not specific due to Dact2 overexpression. All luciferase activities are shown as mean-fold activation compared with the reporter plasmid co-transfected with empty CMV expression plasmid (± SEM from five independent experiments). (TIF) [file pone.0054868.s004.tif]

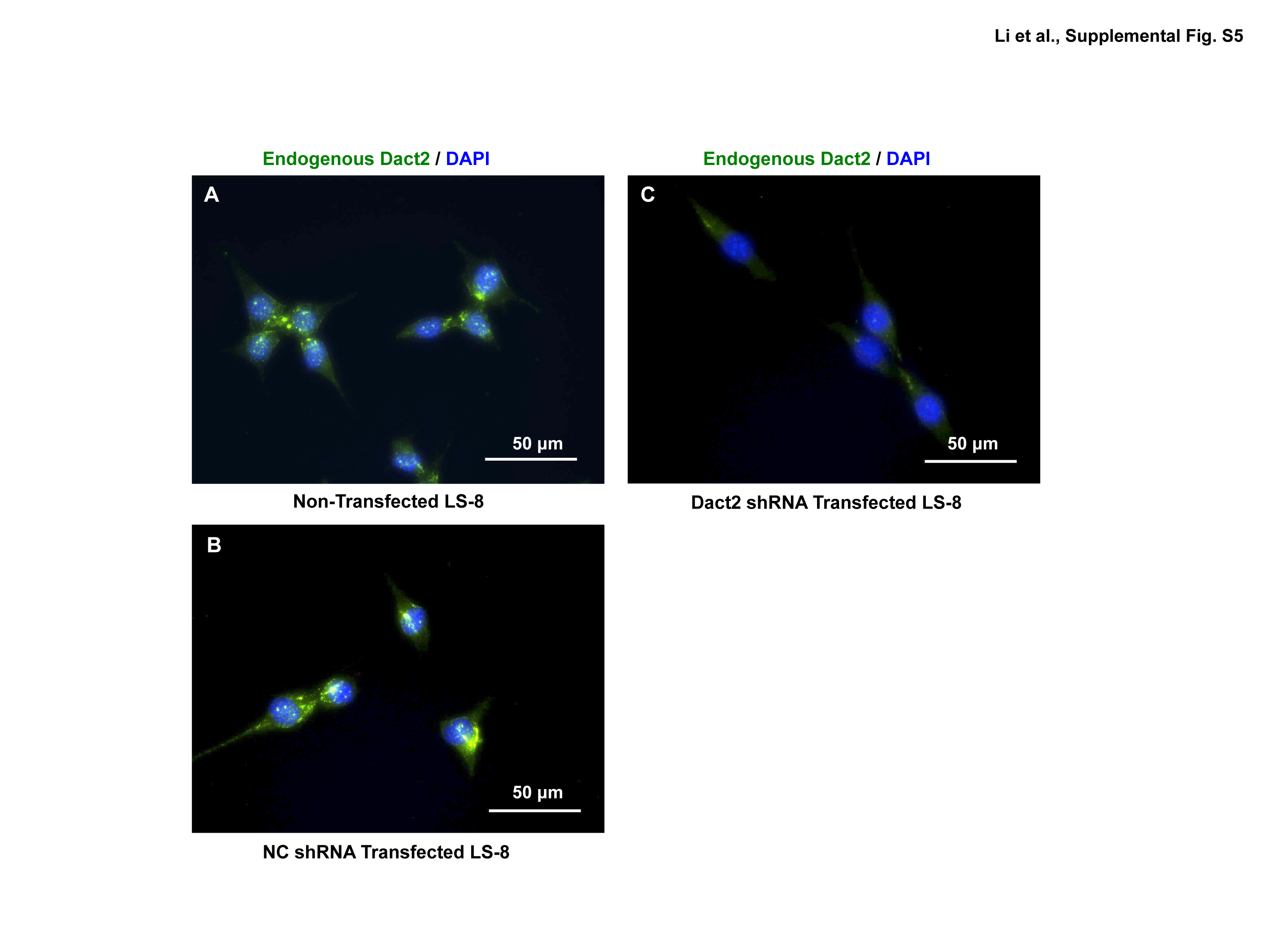

Supplement: Figure S5 — shRNA knocks down Dact2 protein in LS-8 cells. (A) Dact2 protein was probed by Dact2 primary antibody and labeled with FITC in non-transfected LS-8 cells. (B) Similar level of Dact2 protein staining was seen in NC-shRNA transfected LS-8 cells. (C) Dact2 protein staining was significantly lower in Dact2 shRNA transfected LS-8 cells. Results support efficiency of Dact2 shRNA, as well as the specificity of Dact2 antibody in immunocytostaining experiments shown in Fig. 1 and Fig. 6. All cells were counter staining with DAPI to show nuclei. Scale bars represent 50 μm. (TIF) [file pone.0054868.s005.tif]
